# Role of Antioxidants in Modulating the Microbiota–Gut–Brain Axis and Their Impact on Neurodegenerative Diseases

**DOI:** 10.3390/ijms26083658

**Published:** 2025-04-12

**Authors:** Natalia Kurhaluk, Piotr Kamiński, Rafał Bilski, Renata Kołodziejska, Alina Woźniak, Halina Tkaczenko

**Affiliations:** 1 Institute of Biology, Pomeranian University in Słupsk, Arciszewski St. 22 B, 76-200 Słupsk, Poland; halina.tkaczenko@upsl.edu.pl; 2 Department of Medical Biology and Biochemistry, Division of Ecology and Environmental Protection, Collegium Medicum in Bydgoszcz, Nicolaus Copernicus University in Toruń, M. Skłodowska-Curie St. 9, 85-094 Bydgoszcz, Poland; piotr.kaminski@cm.umk.pl; 3 Department of Biotechnology, Institute of Biological Sciences, Faculty of Biological Sciences, University of Zielona Góra, Prof. Z. Szafran St. 1, 65-516 Zielona Góra, Poland; 4 Department of Medical Biology and Biochemistry, Collegium Medicum in Bydgoszcz, Nicolaus Copernicus University in Toruń, M. Karłowicz St. 24, 85-092 Bydgoszcz, Poland; rafal.bilski@cm.umk.pl (R.B.); renatak@cm.umk.pl (R.K.); al1103@cm.umk.pl (A.W.)

**Keywords:** antioxidants, gut microbiota, neurodegeneration, Alzheimer’s disease, Parkinson’s disease, gut–brain axis, therapeutic interventions, microbial metabolites

## Abstract

This narrative review presents the role of antioxidants in regulating the gut microbiota and the impact on the gut–brain axis, with a particular focus on neurodegenerative diseases, such as Alzheimer’s (AD) and Parkinson’s disease (PD). These diseases are characterised by cognitive decline, motor dysfunction, and neuroinflammation, all of which are significantly exacerbated by oxidative stress. This review elucidates the contribution of oxidative damage to disease progression and explores the potential of antioxidants to mitigate these pathological processes through modulation of the gut microbiota and associated pathways. Based on recent studies retrieved from reputable databases, including PubMed, Web of Science, and Scopus, this article outlines the mechanisms by which antioxidants influence gut health and exert neuroprotective effects. Specifically, it discusses how antioxidants, including polyphenols, vitamins, and flavonoids, contribute to the reduction in reactive oxygen species (ROS) production and neuroinflammation, thereby promoting neuronal survival and minimising oxidative damage in the brain. In addition, the article explores the role of antioxidants in modulating key molecular pathways involved in oxidative stress and neuroinflammation, such as the NF-κB, Nrf2, MAPK, and PI3K/AKT pathways, which regulate ROS generation, inflammatory cytokine expression, and antioxidant responses essential for maintaining cellular homeostasis in both the gut and the central nervous system. In addition, this review explores the complex relationship between gut-derived metabolites, oxidative stress, and neurodegenerative diseases, highlighting how dysbiosis—an imbalance in the gut microbiota—can exacerbate oxidative stress and contribute to neuroinflammation, thereby accelerating the progression of such diseases as AD and PD. The review also examines the role of short-chain fatty acids (SCFAs) produced by beneficial gut bacteria in modulating these pathways to attenuate neuroinflammation and oxidative damage. Furthermore, the article explores the therapeutic potential of microbiota-targeted interventions, including antioxidant delivery by probiotics and prebiotics, as innovative strategies to restore microbial homeostasis and support brain health. By synthesising current knowledge on the interplay between antioxidants, the gut–brain axis, and the molecular mechanisms underlying neurodegeneration, this review highlights the therapeutic promise of antioxidant-based interventions in mitigating oxidative stress and neurodegenerative disease progression. It also highlights the need for further research into antioxidant-rich dietary strategies and microbiota-focused therapies as promising avenues for the prevention and treatment of neurodegenerative diseases.

## 1. Introduction

The relationship between the gut microbiota and neurodegenerative diseases is a rapidly developing area of research. It is estimated that the human body contains approximately 100 trillion microbial cells, the majority (approximately 80%) of which are found in the gastrointestinal tract [1]. These microbes perform a wide range of beneficial functions, including digestive, metabolic, immunomodulatory, regulatory, and antagonistic roles [2]. Importantly, the microbiota has been shown to stimulate the regeneration of intestinal epithelial cells and nourish the mucosal lining by producing short-chain fatty acids (SCFAs), which are essential for maintaining gut health [3]. Studies have demonstrated the critical role of SCFAs in supporting intestinal barrier function and modulating immune responses [4].

The exploration of the regulatory potential of the microbiome is becoming increasingly important, particularly in the context of neurodegenerative diseases [5]. According to the World Health Organization (WHO), nearly 50 million people worldwide suffer from dementia, with AD accounting for approximately 75% of these cases [6]. The statistics for other neurological disorders are equally worrying, as the incidence of neurodegenerative diseases continues to rise worldwide [7].

The gut–brain axis is a complex bidirectional communication system linking the gastrointestinal tract and the central nervous system. This axis plays a critical role in maintaining physiological and neurological homeostasis. It involves neural, hormonal, and immunological pathways that regulate brain function, behaviour, and emotional responses [8,9]. Disruptions in this dynamic relationship are increasingly implicated in several health conditions, including neurodegenerative diseases, highlighting the potential role of the gut as a critical mediator of brain health [10].

Recent research has also highlighted the role of the gut microbiota in modulating human behaviour. Studies suggest that up to 90% of serotonin and a significant amount of dopamine, key neurotransmitters involved in mood regulation and cognitive function, are synthesised by gut microbes [11,12]. A deficiency of these substances has been linked to such conditions as chronic pain, insomnia, anxiety, and irritable bowel syndrome (IBS) [13]. Low levels of dopamine have also been observed in schizophrenia and Parkinson’s disease and are associated with an increased risk of developing neurodegenerative diseases, such as AD and PD [14]. Therefore, the composition of the gut microbiota may serve as a potential biomarker of brain health and disease progression [15].

Oxidative stress, defined as an imbalance between the production of reactive oxygen species (ROS) and the body’s ability to neutralise them with antioxidants, is a key mechanism underlying neurodegeneration [16]. In AD and PD diseases, oxidative stress exacerbates neuronal damage by promoting lipid peroxidation, protein misfolding, and DNA damage. This not only accelerates disease progression but also contributes to neuroinflammation, which leads to cognitive and motor dysfunction [17]. This highlights the need for therapeutic approaches that target oxidative stress pathways to mitigate damage and delay disease progression.

Research into the gut–brain axis and its modulation by antioxidants is crucial, as it has far-reaching implications for the understanding and treatment of neurodegenerative diseases and other neurological disorders. Studies in this area could potentially uncover innovative therapeutic strategies that address the underlying causes of these diseases, rather than just treating their symptoms [18,19]. The importance of continuing this research is underlined by the increasing prevalence of neurodegenerative diseases, the role of oxidative stress as a common pathway in neurodegeneration, the impact on non-neurodegenerative diseases, and the promising potential of antioxidants in therapeutic interventions [20].

The prevalence of AD and related dementias is high in people aged 65 years and older, and the global burden is projected to increase dramatically in the coming decades [21]. Similarly, PD is becoming a growing concern in economically developed countries, where its prevalence has reached significant levels in people aged 60–65 years and older [22]. These trends highlight the urgent need for a better understanding of the mechanisms underlying these neurodegenerative diseases, particularly given their significant societal and economic impact [23].

AD is a chronic progressive neurodegenerative disorder characterised by three main groups of symptoms. The first group includes cognitive dysfunction, such as memory impairment, language difficulties, and executive dysfunction, including loss of higher-order planning and intellectual coordination. The second group includes psychiatric symptoms and behavioural disturbances, often referred to as non-cognitive symptoms, such as depression, hallucinations, delusions, and agitation. The third group includes difficulties with activities of daily living, ranging from instrumental activities (e.g., driving and shopping) to basic activities (e.g., dressing and eating independently) [24,25]. Early diagnosis and intervention remain critical due to the progressive nature of these symptoms.

As demonstrated in the following references, the neuronal damage and disease progression of AD [26], PD [27], Huntington’s [28], and ALS [29,30] is driven by oxidative stress, resulting from an excess of ROS overwhelming the body’s antioxidant defences, leading to lipid peroxidation, protein misfolding, and DNA damage [31,32]. Research into antioxidant strategies provides a unified approach to targeting this common pathological mechanism. The potential for early intervention remains significant, especially considering that gut microbiota dysbiosis and oxidative stress often precede clinical symptoms in neurodegenerative diseases [33]. Understanding these early changes may facilitate the development of preventive therapies that improve long-term outcomes.

Furthermore, the implications for non-neurodegenerative diseases are equally significant. Beyond neurodegeneration, oxidative stress and gut dysbiosis have been implicated in other nervous system injuries, such as traumatic brain injury [34] and multiple sclerosis (MS) [35]. Insights gained from studies of the gut–brain axis may offer therapeutic benefits for a wider range of neurological disorders, thus widening the scope of potential interventions.

Antioxidants have been shown to exert a neuroprotective effect by reducing ROS, modulating inflammation, and supporting neuronal survival [36]. Research into the influence of antioxidants through the gut microbiota represents a novel and holistic approach to neuroprotection [37]. While antioxidant strategies cannot completely eliminate the risk of neurodegenerative disease, they have emerged as an effective means of reducing oxidative stress, increasing neuronal resilience, and delaying disease onset [17]. The integration of antioxidant interventions with lifestyle modifications and personalised approaches has led to the development of comprehensive preventive strategies that effectively address the multifactorial nature of neurodegenerative diseases [38,39].

The primary objective of this review is to explore the role of antioxidants in modulating the gut microbiota and their therapeutic potential in the context of the gut–brain axis. Through an analysis of recent studies, this review will highlight the impact of antioxidants on the gut microbial composition, reduction in oxidative stress, and modulation of key molecular pathways associated with neurodegeneration. Specifically, the review aims to analyse current evidence on the contribution of the gut microbiota to the pathogenesis of AD and PD diseases, identify key mechanisms by which the gut microbiota interacts with the brain (focusing on microbial metabolites, the immune system, neurotransmitter synthesis, and hormonal pathways), and evaluate therapeutic strategies that exploit modulation of the gut microbiota (including dietary interventions, probiotics, and other microbiome-based approaches) as innovative methods for disease prevention and management.

This work underscores the critical need to address the growing global prevalence of neurodegenerative diseases by advancing our understanding of the gut–brain axis. By systematically synthesising evidence from a wide range of studies, this review aims to provide novel insights into the development of strategies to mitigate oxidative damage and improve outcomes in neurodegenerative diseases.

The novelty and relevance of our review lies in its focus on the emerging multidisciplinary field of study, synthesising current evidence on the role of gut microbiota in neurodegenerative diseases. In doing so, it highlights not only the complexity of this global health challenge but also innovative avenues for the development of preventive and therapeutic strategies. Addressing these issues is critical to reducing the growing global burden of AD and PD disease.

## 2. Materials and Methods

The comprehensive literature review conducted for this study included several reputable databases to ensure exhaustive coverage of current knowledge and evidence. The following databases were searched: PubMed, Cochrane Library, Medline, Embase, SciSearch, Web of Science, Scopus, Google Scholar, and relevant conference proceedings. The inclusion criteria focused on peer-reviewed articles, systematic reviews, and meta-analyses published in English and addressing key topics, such as the role of the gut microbiota in neurodegenerative diseases (particularly AD and PD), mechanisms underlying the gut–brain axis (including microbial metabolites, immune modulation, and neurotransmitter synthesis), and potential therapeutic approaches targeting the gut microbiota to mitigate neurodegeneration.

The articles were selected based on their relevance to the parameters, including studies investigating correlations between the gut microbiota composition and neurodegenerative conditions, research evaluating the biomarkers of microbiota disruption and their association with disease progression, and investigations of the efficacy of interventions (such as probiotics, dietary changes, and gut microbiota modulation) on cognitive and behavioural outcomes.

### 2.1. Gut–Brain Axis and Its Bidirectional Regulation

**Figure 1:**
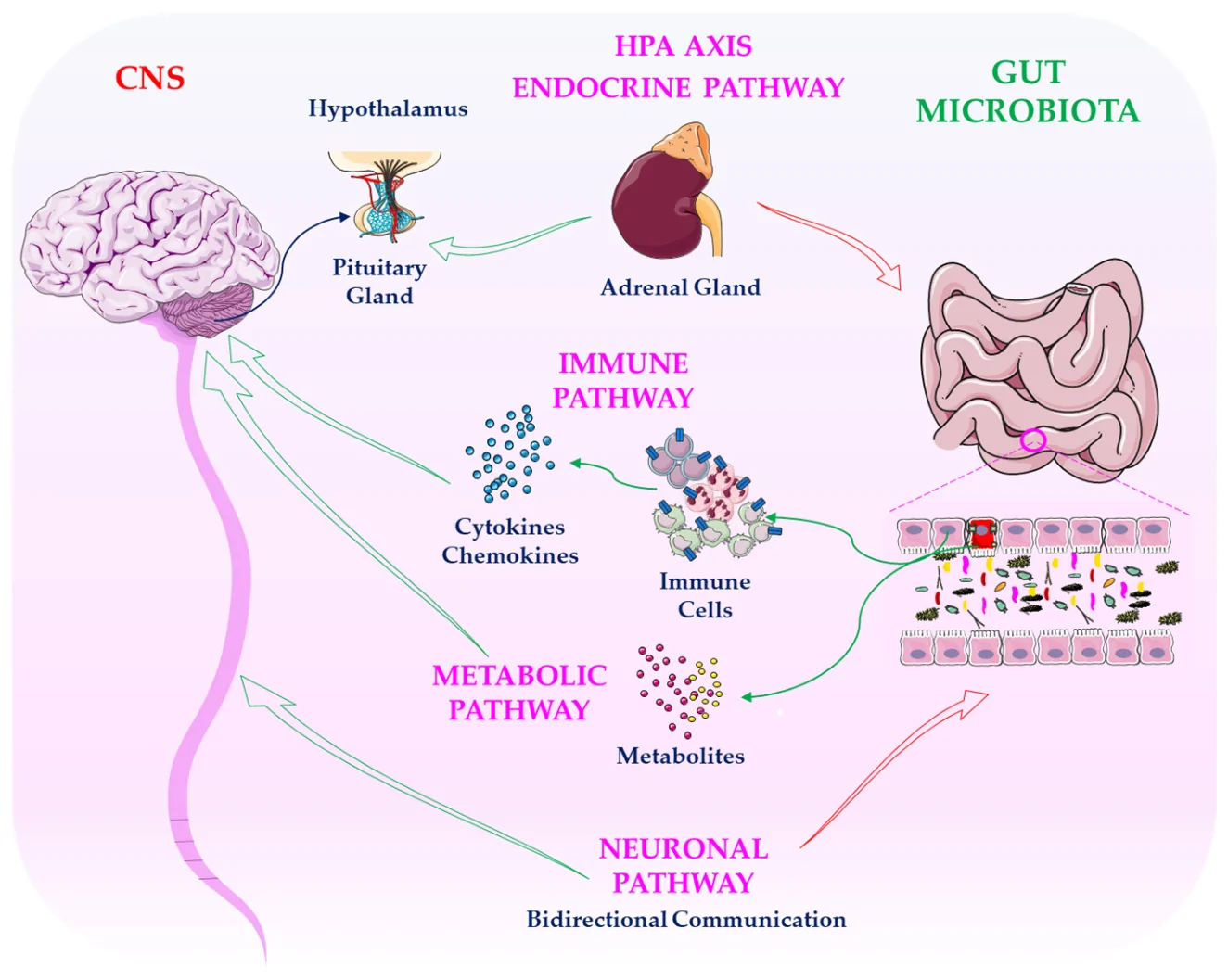
Bidirectional communication in the microbiome–brain axis. Bidirectional signalling within the microbiome–brain axis involves complex neural, endocrine, immune, and metabolic pathways, enabling dynamic crosstalk between the gut microbiota and the central nervous system. These interactions influence neurodevelopment, behaviour, and host physiology, highlighting the therapeutic potential of microbiota-targeted interventions in neurodegenerative diseases. Image provided by Servier Medical Art (https://smart.servier.com), licensed under CC BY 4.0 (https://creativecommons.org/licenses/by/4.0/, accessed on 26 March 2026). Abbreviations: CNS—central nervous system; HPA Axis—hypothalamic–pituitary–adrenal axis.

The gut–brain axis is a complex system of communication between the gastrointestinal tract and the brain, involving multiple signalling pathways. This bidirectional communication, facilitated by neural, hormonal, and immune mechanisms, is known as the “microbiota–gut–brain axis” [9,40]. The gut microbiota and its metabolites have the capacity to influence both immune responses and neurological processes through their connection to the central nervous system (CNS) [41]. This concept has been extensively studied in relation to the development of functional gastrointestinal disorders, such as irritable bowel syndrome (IBS) [42]—often associated with stress—and its role in the pathophysiology of depression [43,44], autism [45,46], and neurodegenerative diseases [47]. These dependencies are shown in Figure 1.

The axis comprises several interconnected physiological systems and structures, including the CNS, the enteric nervous system (ENS), and the gut microbiota. It is a bidirectional conduit that allows signals to flow between these systems, allowing the CNS to modulate the motor, sensory, and secretory functions of the gastrointestinal tract (GI) tract and vice versa [8]. This link is regulated at several levels: (a) nervous level—mainly via the vagus nerve or spinal cord [48], (b) metabolic level—via metabolites, such as short chain fatty acids (SCFAs) and tryptophan [49,50], (c) immune level—via cytokines and chemokines, and (d) endocrine level—mainly through the hypothalamic–pituitary–adrenal (HPA) axis [51].

Another critical aspect of this axis is microglial activation and neuroinflammation mediated by cytokines and chemokines. The gut microbiota has been shown to play a role in regulating the release of cytokines and chemokines, which are signalling molecules that activate microglia in the CNS [52,53]. Chronic microglial activation is a hallmark of neurodegenerative disease. The activation of microglia, the resident immune cells of the brain, is central to neuroinflammation [54]. Gut-derived signals, such as bacterial products or metabolites, can trigger microglial activation, leading to chronic inflammation in the CNS and contributing to neuronal damage in such diseases as AD and PD [55,56]. Prolonged microglial activation leads to the release of neurotoxic substances, further promoting neuronal damage and disease progression [57].

The integrity of the blood–brain barrier (BBB) is another critical factor in these processes. The gut microbiota influences BBB permeability through the production of metabolites, such as SCFAs, and inflammatory mediators [49,58]. A compromised BBB allows toxins and inflammatory agents to enter the brain, contributing to the progression of neurodegenerative diseases [59]. Zonulin, a protein that regulates BBB permeability, is a key player in this context [60]. Impaired microbiome composition can alter zonulin expression, increasing BBB permeability and allowing neurotoxic substances to enter the brain. This increased permeability is associated with neuroinflammation and the development of neurodegenerative diseases [61].

Thus, the gut–brain axis plays a critical role in the development and progression of neurodegenerative diseases. By modulating the gut microbiota, it may be possible to influence brain function, reduce neuroinflammation, and potentially develop therapeutic options for the treatment of such diseases as AD, PD, and other neurodegenerative disorders [19]. Understanding the molecular mechanisms involved in this interaction is essential for developing targeted therapies that harness the gut–brain connection to improve neurological health.

### 2.2. Role of the Vagus Nerve in the Microbiota–Gut–Brain Axis and Its Implications for Neurodegenerative Diseases

**Figure 2:**
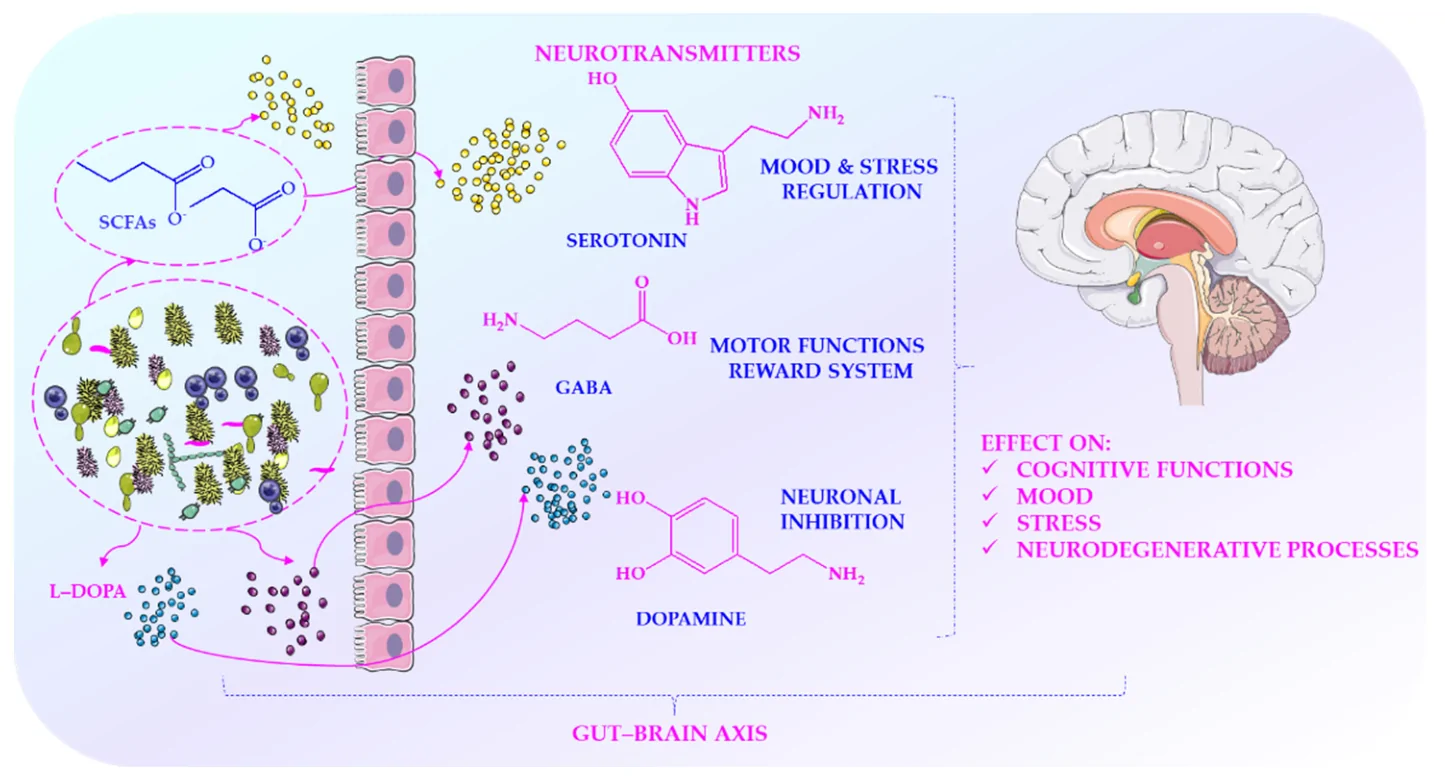
Gut Microbiota–mediated modulation of neurotransmitter synthesis and its impact on brain function and neurodegeneration. Role of the gut microbiota in regulating the synthesis and metabolism of key neurotransmitters (serotonin, dopamine, and GABA) and its impact on mood, cognitive function, and neurodegenerative processes. Alterations in gut microbial composition may disrupt the gut–brain axis, contributing to mood disorders and the progression of neurological diseases. Image provided by Servier Medical Art (https://smart.servier.com), licensed under CC BY 4.0 (https://creativecommons.org/licenses/by/4.0/, accessed on 26 March 2026). Abbreviations: GABA—gamma-aminobutyric acid; L-DOPA—L-3,4-dihydroxyphenylalanine; SCFAs—short-chain fatty acids.

The gut microbiota plays an essential role in modulating the synthesis and metabolism of several key neurotransmitters, including serotonin, dopamine, and GABA, which regulate mood, cognition, and stress responses [62]. Approximately 90% of serotonin is produced in the gut, where microbial communities influence its production [63]. Alterations in the gut microbiota can lead to imbalances in serotonin levels, contributing to mood disorders, such as depression and anxiety—conditions often associated with neurodegenerative diseases [64]. Dopamine, essential for reward processing and motor function, is also synthesised in the gut and its dysregulation is implicated in Parkinson’s disease, where dopamine-producing neurons degenerate [65]. GABA, an inhibitory neurotransmitter, is also affected by the gut microbiota, affecting brain excitability and potentially contributing to neurodegeneration [11,66]. Thus, changes in the composition of the gut microbiota can have a significant impact on mood, cognition, and the progression of neurological disease, as shown in Figure 2.

The interaction between the gut microbiota and the CNS is primarily mediated by the vagus nerve (VN), as shown by Haney et al. (2018) and Bonaz et al. (2017), which serves as a critical link between the two systems [67,68]. The vagus nerve is a mixed nerve consisting of 80% afferent and 20% efferent fibres and is responsible for transmitting signals from peripheral organs and the microbiota to the CNS [68]. These signals influence behaviour, emotions, and cognitive functions, which in turn trigger a response from the nervous system that can be either adaptive or maladaptive [67]. Maladaptive responses contribute to the development of gastrointestinal pathology and neurological disorders, including autism spectrum disorders, AD, and PD [67].

Bonaz et al. (2017) conducted a study focusing on the bidirectional communication within the microbiota–gut–brain axis, with a particular focus on the VN as a critical mediator [68]. The VN plays a key role in sensing microbial metabolites in the gut and transmitting this information to the CNS, where it is integrated into the central autonomic network. The VN also modulates inflammation through its cholinergic anti-inflammatory pathway, alleviating peripheral inflammation and reducing intestinal permeability, which influences the composition of the gut microbiota. Stress impairs vagal function, leading to disturbed microbial ecology and contributing to gastrointestinal disorders, such as irritable bowel syndrome (IBS) and inflammatory bowel disease (IBD), both of which are characterised by low vagal tone and increased inflammatory responses [69,70].

Butt et al. (2020) investigated the anatomical basis of transcutaneous auricular vagus nerve stimulation (taVNS) and highlighted its therapeutic potential [71]. The study revealed the anatomical pathways that are accessible for non-invasive stimulation, particularly the auricular branch. A precise anatomical understanding of taVNS is crucial to improve its efficacy and specificity in modulating autonomic functions [71]. Bonaz et al. (2018) further explored the role of the VN in the microbiota–gut–brain axis, demonstrating its essential involvement in bidirectional communication between the gut microbiota and the CNS. This has important implications for the treatment of neuroinflammation and altered gut microbiota and supports vagus nerve modulation as a therapeutic strategy for disorders of gut–brain homeostasis [72].

The cholinergic anti-inflammatory pathway was identified as a key mechanism in the management of gastrointestinal diseases, such as IBD, in a study investigating the role of the VN in the neuroimmune axis [68]. Similarly, Hesampour et al. (2024) and Cirillo et al. (2022) investigated both invasive and non-invasive vagus nerve stimulation (VNS), advocating personalised approaches to the management of Crohn’s disease and other IBDs [73,74]. Evidence suggests a reduction in inflammation and improved gut barrier integrity following VNS, which is further supported by studies conducted by Bonaz (2022) and Bonaz et al. (2013), highlighting the anti-inflammatory properties of VNS, particularly in repairing gut barrier dysfunction [75,76]. Taken together, these studies position VNS as a transformative therapeutic modality that harnesses its neuroimmune and cholinergic pathways to treat chronic gastrointestinal and systemic inflammatory conditions.

Thus, the VN plays a central role in maintaining homeostasis between the gastrointestinal system and the brain, detecting microbial signals and modulating inflammation via the anti-inflammatory pathway. However, stress-induced suppression of vagal activity disrupts this balance, leading to increased inflammation, impaired microbiome composition, and manifestation of gastrointestinal disorders, such as IBS and IBD. Therapeutic strategies, including vagal nerve stimulation, offer promising potential to restore the balance within the microbiota–gut–brain axis and alleviate these conditions [48].

The understanding of alternative pathways of interaction between the gut microbiota and the CNS has led to the hypothesis that the VN is a critical mediator between these systems. The regulatory influences are bidirectional, extending from the CNS to the gut and vice versa [77]. Research has explored the microbiota effects on the intestinal wall and the VN, highlighting the rich innervation of the gastrointestinal tract (GIT). The VN modulates both the activity of the microbiota and the integrity of the intestinal wall [48,72]. In normal conditions, the VN exerts its effects through both direct and indirect mechanisms: direct innervation, where it innervates and coordinates the function of smooth muscle cells and intramural ganglia in the gut wall, and paracrine actions, where the VN releases acetylcholine and neuroactive modulators that affect other components of the gut environment, including immune cells in the submucosa. These cells are influenced by vagal activity, which may help maintain the integrity of the epithelial barrier and modulate immune responses, particularly in altered gut microbiota or pathological states [48]. Studies have also demonstrated the anti-inflammatory properties of vagal activation, which can suppress inflammatory responses, highlighting its therapeutic potential in conditions involving intestinal inflammation [78]. This complex interplay between the VN, the gut microbiota, and the intestinal wall provides insights into mechanisms for maintaining intestinal and systemic homeostasis [79].

Enterochromaffin-like cells and APUD cells are also influenced by paracrine signals, which may contribute to maintaining the intestinal barrier and modulating inflammatory responses, especially in microbial disarray or other pathological conditions [80,81]. Research has also demonstrated anti-inflammatory effects of VN activation [75,82]. By inhibiting the activity of inflammatory cells, the VN can suppress inflammatory responses, suggesting its potential therapeutic importance in conditions involving intestinal inflammation [83,84]. The complex interactions between the VN, the gut microbiota, and the intestinal wall underline the complexity of these relationships and provide insights into potential mechanisms for maintaining intestinal and systemic homeostasis [48].

On the other hand, the VN-independent influence of the gut microbiota on the CNS is also mediated by the immune system, as shown by Banks et al. (1995) [85]. This influence is stimulated by cytokines that circulate and affect the CNS. This link is evident in such syndromes as anorexia, anhedonia, reduced pain thresholds, and slowed psychomotor functions [86]. It has been shown that microbial infection-induced activation of the immune system in the gut disrupts the intestinal barrier, which activates the enteric nervous system and alters sensory and motor functions [87]. These changes amplify interoceptive signals, increasing pain and anxiety [86].

An important component of the parasympathetic nervous system, the VN is the longest cranial nerve, which acts as a crucial bidirectional conduit between the body and the brain, primarily contributing to homeostasis [71]. The origin of this nerve is located in the brainstem, from which it extends to the proximal two-thirds of the colon, thereby innervating a significant number of thoracic and abdominal viscera. It is a mixed nerve, predominantly composed of afferent fibres, with a smaller proportion of efferent fibres [72]. In neurodegenerative diseases, disruptions in this pathway—which are frequently associated with alterations in gut microbial composition—contribute to increased oxidative stress, neuronal dysfunction and inflammation, which are key hallmarks of neurodegeneration [72,88].

The VN, a key player in the transmission of signals between the gastrointestinal tract and the brain, is linked to the immune and endocrine systems, underlining its importance in these processes [89]. Microbial products, such as SCFAs, that cross the BBB influence brain function and maintain BBB integrity. Disruption of this communication, typically caused by microbial disarray, can lead to neuroinflammation and neuronal dysfunction, contributing to neurodegenerative diseases [49,90]. As the VN is a critical component of the gut–brain axis, gut microbial dysregulation can disrupt this pathway, affecting the CNS response to inflammation and stress and exacerbating neurodegenerative processes [48].

### 2.3. Critical Role of Gut-Derived Metabolites in Neuroinflammation and Blood–Brain Barrier Integrity

**Figure 3:**
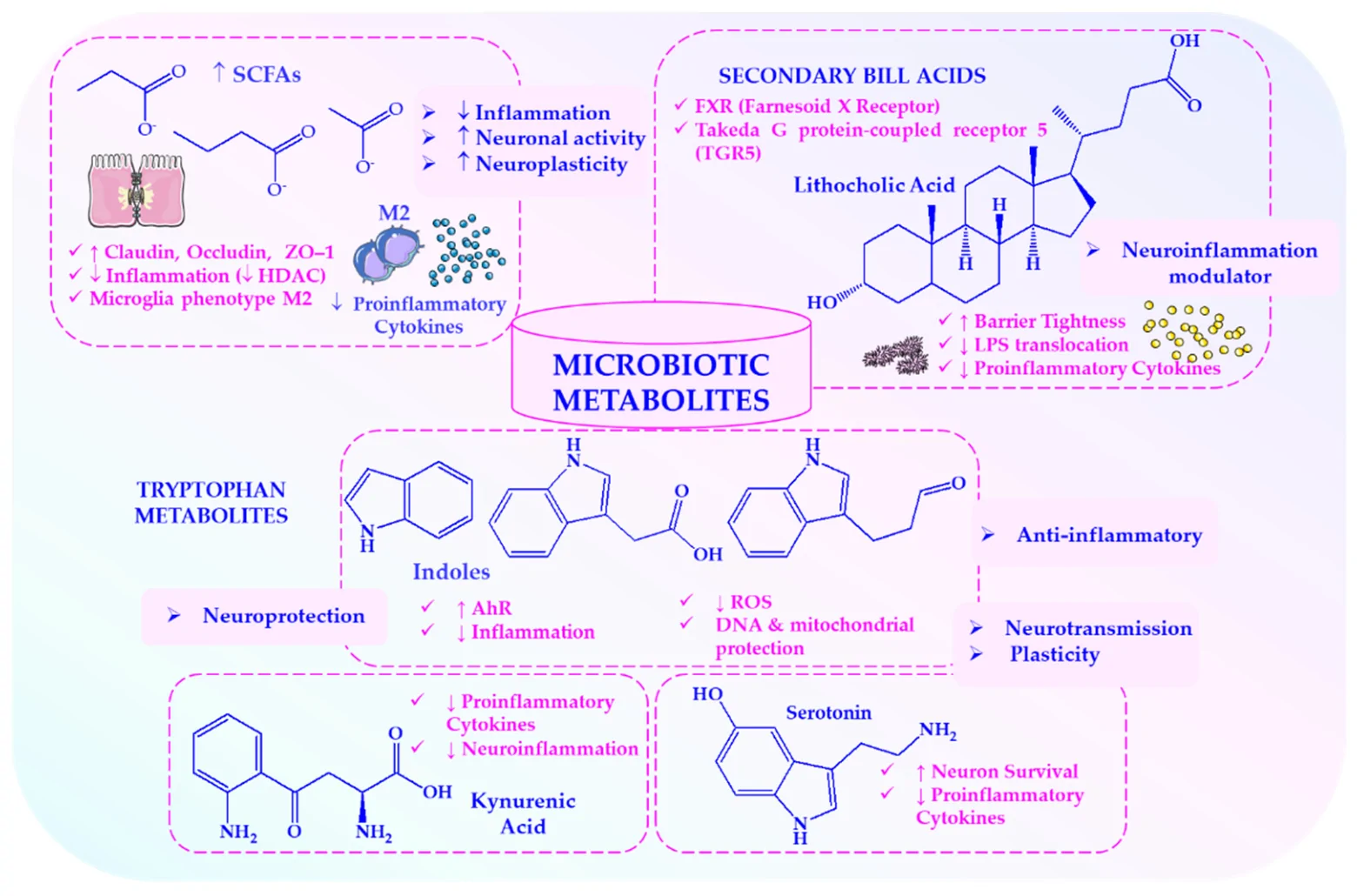
Metabolic pathways of the gut microbiota and their relevance to neurophysiology. Interactions between key microbial metabolic pathways include the role of short-chain fatty acids (SCFAs) in neurotransmission, tryptophan metabolism via the kynurenine pathway, and bile acid and lipid metabolism. Microbial metabolites such as SCFAs, tryptophan derivatives, and bile acids influence brain function through complex metabolic and signaling mechanisms, modulating neuroinflammation, immune responses, and the gut–brain axis. Image provided by Servier Medical Art (https://smart.servier.com), licensed under CC BY 4.0 (https://creativecommons.org/licenses/by/4.0/, accessed on 26 March 2026). Abbreviations: AhR—aryl hydrocarbon receptor; DNA—deoxyribonucleic acid; FXR—farnesoid X receptor; HDAC—histone deacetylases; LPS—lipopolysaccharide; M2—M2 macrophages; ROS—reactive oxygen species; SCFAs—short-chain fatty acids; TGR5—Takeda G protein-coupled receptor 5; ZO-1—zonula occludens-1; ↑—increase/upregulation; ↓—decrease/downregulation.

Gut-derived metabolites play a particularly important role in gut–brain function and communication. Among these, short-chain fatty acids (SCFAs), produced by gut bacteria during the fermentation of dietary fibre, are critical in regulating neuroinflammation and maintaining brain homeostasis [49,91]. For example, butyrate has been shown to suppress pro-inflammatory cytokines and improve BBB function, thereby protecting the brain from neuroinflammatory damage [92,93]. SCFAs also modulate the expression of genes involved in the immune response, reducing the systemic inflammatory burden that contributes to neurodegenerative disease [94,95]. In addition, SCFAs influence neurotransmitter synthesis and neuronal plasticity, which are essential for cognitive function and mental health [49,96]. The metabolic and microbial metabolite pathways are illustrated in Figure 3.

SCFAs, such as butyrate, acetate, and propionate, have anti-inflammatory effects and support BBB integrity [91]. However, gut microbial imbalance can lead to reduced SCFA production, increased BBB permeability, and increased neuroinflammation [97]. These changes have been implicated in the pathogenesis of neurodegenerative diseases, such as AD and PD [98,99].

The role of the microbiota in the regulation of systemic and neuroinflammation has been extensively discussed in the scientific literature [55,97,100]. It is well established that the gut microbiota plays a pivotal role in modulating both systemic and neuroinflammatory responses through cytokine signalling and its influence on gut-associated lymphoid tissue (GALT) [101]. GALT is a critical component of immune homeostasis and its disruption, such as in disturbed microbial ecology, can lead to excessive production of pro-inflammatory cytokines [102]. Cytokines, such as interleukin-6 (IL-6), interleukin-1β (IL-1β), and tumour necrosis factor-alpha (TNF-α), can cross the BBB, exert direct effects on neuronal function, and contribute to neuroinflammatory changes in the brain [103,104]. Thus, the gut microbiota exerts broad regulatory effects on inflammation, influencing not only peripheral immune responses but also CNS function.

**Figure 4:**
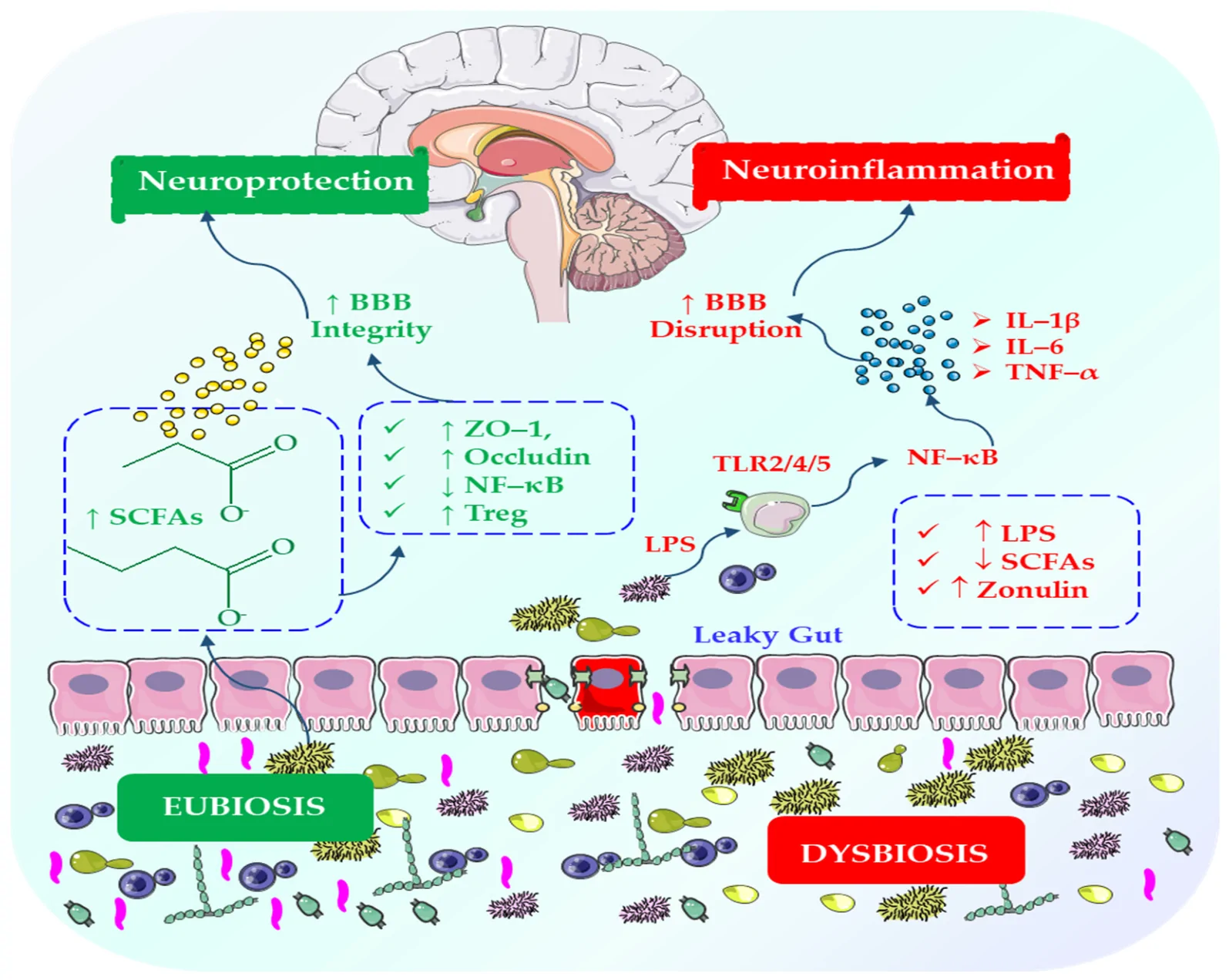
Immune system pathways. The gut microbiota play a crucial role in modulating both systemic and neuroinflammatory responses. The influence of the gut microbiota on immune signalling is achieved through cytokine production, regulation of blood–brain barrier (BBB) permeability, and interactions with key immune biomarkers and molecular pathways. These pathways underscore the intricate communication between gut-resident microbes, immune cells, and the central nervous system, thereby underscoring the significance of microbiota in maintaining immune homeostasis and potentially contributing to the pathophysiology of neurological and inflammatory disorders. Image provided by Servier Medical Art (https://smart.servier.com), licensed under CC BY 4.0 (https://creativecommons.org/licenses/by/4.0/, accessed on 26 March 2026). Abbreviations: BBB—blood—brain barrier; IL-1β—interleukin-1 beta; IL-6—interleukin-6; LPS—lipopolysaccharide; NF-κB—nuclear factor kappa-light-chain-enhancer of activated B cells; SCFAs—short-chain fatty acids; TLR 2/4/5—Toll-like receptors 2, 4, and 5; TNF-α—tumor necrosis factor alpha; Treg—regulatory T cells; ZO-1—zonula occludens-1, ↑—increase/upregulation; ↓—decrease/downregulation.

Cytokine-mediated signalling associated with gut microbiota disruption results in elevated levels of pro-inflammatory cytokines, which can cross the BBB and alter neuronal function [58]. Activation of inflammatory pathways involves key molecules, such as lipopolysaccharides (LPS) and Toll-like receptors (TLRs), which trigger inflammatory cascades, including activation of the NF-κB pathway [105,106]. Biomarkers indicative of these processes include (a) faecal calprotectin levels, a marker of gut inflammation [107], (b) serum IL-6 levels, indicative of systemic inflammation [108], and (c) neuroinflammatory markers, such as increased S100B protein levels, associated with glial cell damage and BBB dysfunction [109,110]. These biomarkers, combined with changes in the microbiota composition, provide valuable diagnostic insights into systemic and neuroinflammatory activity (Figure 4).

The gut microbiota has a profound influence on BBB integrity, a critical factor in neuroinflammatory processes. Microbial metabolites, particularly SCFAs, such as butyrate, acetate, and propionate, contribute to BBB maintenance by regulating the activity of tight junction proteins, including zonula occludens-1 (ZO-1), occludin, and claudins [91,111]. Unbalanced gut flora-induced reductions in SCFA production can weaken the BBB, increasing its permeability and allowing harmful substances to enter the brain. This increased permeability is not only a hallmark of neuroinflammatory disorders but also a key risk factor for the development of neurodegenerative diseases, such as AD and PD [112,113]. Zonulin, a regulator of intestinal permeability, has also been implicated in BBB dysfunction. Elevated levels of zonulin are associated with impaired barrier integrity, linking intestinal permeability to neuroinflammatory processes [60,114]. Similarly, S100B protein, which reflects glial cell damage, serves as an additional biomarker of BBB disruption and neuroinflammation [109].

Toll-like receptors (TLRs) play a crucial role in innate immunity by recognising pathogen-associated molecular patterns (PAMPs) and initiating immune responses. While most TLRs are expressed in haematopoietic immune cells, they have also been detected in non-immune tissues, including adipocytes [115]. In animal models, 12 members of the TLR family have been identified, although only 10 have been extensively studied. Of these, TLR2, TLR4, and TLR5 have been specifically implicated in metabolic and immune processes in human adipocytes [116]. For example, TLR2 is activated by saturated fatty acids and bacterial peptidoglycans during endotoxaemia. Interestingly, TLR2 deficiency has been associated with reduced synthesis of inflammatory mediators, reduced macrophage infiltration into white adipose tissue, and reduced risk of obesity-related inflammation [117]. Overactivation of TLRs due to gut dysbiosis can lead to excessive production of inflammatory mediators, which can fuel neuroinflammatory processes. Chronic dysregulated immune signalling in the gut can propagate systemic inflammation, ultimately affecting the brain and accelerating neurodegenerative processes [118,119].

Thus, the gut microbiota plays a multifaceted role in regulating systemic and neuroinflammation through cytokine signalling, BBB modulation, and immune activation via TLR pathways. Perturbations in microbial homeostasis can promote inflammatory responses that not only affect peripheral immunity but also contribute to CNS dysfunction and the pathogenesis of neurodegenerative diseases [120]. Understanding these mechanisms provides a basis for potential therapeutic interventions aimed at modulating the gut microbiota to reduce inflammation and maintain neurological health.

### 2.4. Gut Microbiota as Mediators of Endocrine Pathways, Hormonal Communication, and Neuroinflammation

**Figure 5:**
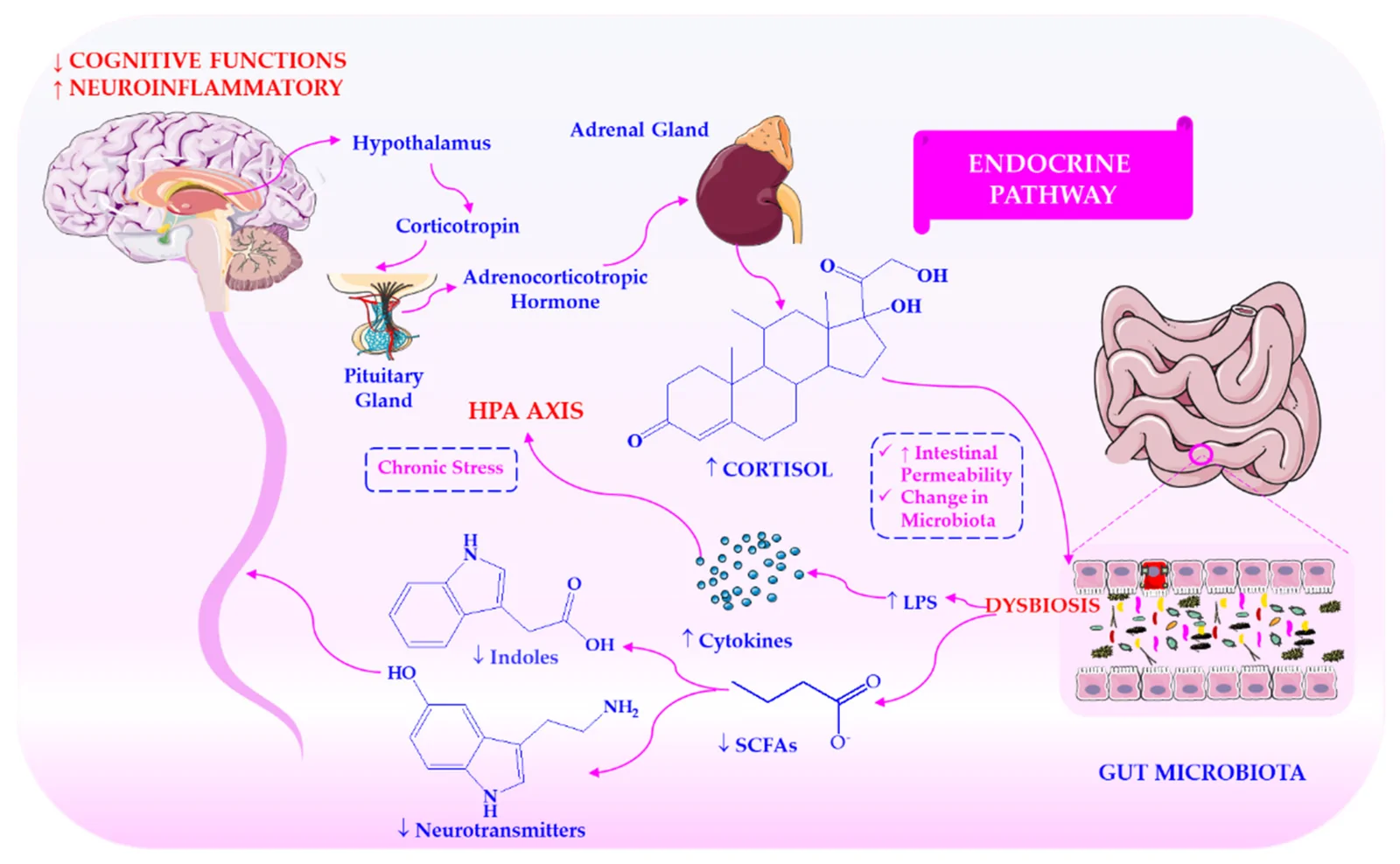
Gut microbiota in the regulation of the HPA axis and neuroendocrine signaling. The gut microbiota modulates the hypothalamic–pituitary–adrenal (HPA) axis, influencing stress, appetite, and behavior. Intestinal microorganisms regulate cortisol and ACTH secretion and synthesize neurotransmitters, as well as cytokines and indoles, thereby affecting mood and cognitive functions. Disruption of gut homeostasis may lead to HPA axis dysregulation and hormonal imbalance. Image provided by Servier Medical Art (https://smart.servier.com), licensed under CC BY 4.0 (https://creativecommons.org/licenses/by/4.0/, accessed on 26 March 2026). Abbreviations: HPA Axis—hypothalamic–pituitary–adrenal axis; LPS—lipopolysaccharides; SCFAs—short-chain fatty acids, ↑—increase/upregulation; ↓—decrease/downregulation.

The role of the gut microbiota in endocrine pathways and hormonal communication has been the subject of numerous studies demonstrating its significant influence on hormonal regulation [121]. These pathways shape key physiological processes related to stress, appetite, and behaviour through the HPA axis, a complex bidirectional communication network linking the central nervous system and the gut [51]. Microbial-derived signals modulate endocrine responses, and perturbations in gut homeostasis can lead to dysfunction in this regulatory network [66,122]. Dysregulation of the HPA axis—often induced by gut dysfunction—has been shown to contribute to hormonal imbalances that affect not only stress responses but also emotional and cognitive functions [51]. The role of the microbiota in these processes includes regulation of cortisol secretion, modulation of adrenocorticotropic hormone (ACTH) levels, and interaction with neuroendocrine signalling pathways (Figure 5).

The gut microbiota plays a critical role in the synthesis of neurotransmitters that affect mood regulation and cognitive function [11]. Gut bacteria have been shown to contribute to the production of key neurotransmitters, including serotonin (5-HT), dopamine, norepinephrine (NE), and gamma-aminobutyric acid (GABA) [12]. These molecules regulate various physiological functions and rely on such pathways as the metabolism of tryptophan, a precursor to serotonin, and the kynurenine pathway, which links microbial activity to brain function [50]. SCFAs produced by gut microbes also modulate neurotransmitter activity [49]. Biomarkers of these interactions include serotonin precursor levels, fluctuations in gut-derived dopamine production, and variations in SCFA concentrations, all of which provide insight into the microbiota impact on neuroendocrine health [11].

Such biomarkers as salivary cortisol and ACTH levels serve as indicators of HPA axis activity [123], while changes in serotonin precursors and gut-derived dopamine reflect microbial contributions to mood and cognition [65]. Modulation of the HPA axis is a central component of the stress response system, resulting in increased secretion of cortisol and upstream regulators, such as ACTH and corticotropin-releasing factor [124]. Elevated levels of these hormones can alter physiological stress responses, potentially exacerbating stress-related disorders. In addition, cytokines associated with stress and immune response, such as IL-6 and TNF-α, are modulated by microbial activity [125]. The interplay between microbial metabolites and immune signalling may contribute to neuroinflammation, further emphasising the role of the gut microbiota in stress-related conditions [126].

The function of neurons is modulated primarily by microbiota-derived metabolites through the process of oxidative stress, which is a key mechanism in neurodegenerative disorders [17,127,128]. Gut microbial disarray has been associated with elevated nitric oxide (NO) levels, which, when present in excess, have been shown to exacerbate oxidative stress and neuronal injury [17,129]. Conversely, short-chain fatty acids (SCFAs) have been shown to exhibit neuroprotective effects by promoting glutathione synthesis and reducing mitochondrial ROS generation, thereby preserving neuronal integrity [113,130].

The second humoral pathway involves microbiota-derived LPS, which can affect gene expression in neural tissue [131]. In cases of intestinal microbial dysregulation or increased intestinal permeability (as seen in inflammatory bowel disease), elevated levels of LPS are detected in the bloodstream. This suggests a possible link between microbial endotoxins and neuroinflammatory responses [132]. LPS has been shown to interact with regulatory regions of chromatin in neuronal nuclei, disrupting normal genomic function [133]. Studies suggest that LPS exposure can reduce mRNA synthesis and downregulate the expression of neurofilament light chain (NF-L), a key structural component of the neuronal cytoskeleton [131,134]. This disruption of neuronal integrity has been linked to synaptic loss and cytoarchitectural perturbations similar to those observed in neurodegenerative diseases [131].

Thus, the gut microbiota plays a fundamental role in modulating endocrine pathways and hormonal communication, significantly influencing physiological processes, such as stress response, appetite regulation, and behaviour. Through bidirectional communication via the hypothalamic–pituitary–adrenal axis, the gut microbiota modulates endocrine signalling, with altered gut microbiota leading to hormonal imbalances and altered neurochemical homeostasis [62]. In addition, microbial metabolites contribute to neurotransmitter synthesis, oxidative stress regulation, and gene expression in neural tissue, further implicating the microbiota in neuroinflammation and neurodegenerative diseases. Understanding these mechanisms may provide novel therapeutic strategies for stress-related behavioural and cognitive disorders.

The gut microbiota plays a critical role in the synthesis and metabolism of key neurotransmitters, including serotonin and dopamine [135]. Imbalances in these neurotransmitters can significantly affect mood, cognitive function, and motor skills and contribute to the pathogenesis of neuropsychiatric and neurodegenerative disorders, such as depression, anxiety, and PD [14,136]. One of the critical pathways linking the gut microbiota to neurotransmitter regulation is tryptophan metabolism. Tryptophan, an essential dietary amino acid, serves as a precursor for several bioactive compounds, including serotonin and kynurenine [50,137]. The gut microbiota modulates tryptophan metabolism by influencing the balance between the serotonin and kynurenine pathways [50,138,139]. Alterations in these pathways have been associated with neuropsychiatric symptoms and neurodegenerative diseases [137]. Reduced serotonin synthesis due to gut microbial imbalance can negatively affect mood regulation and cognitive processes, while increased kynurenine pathway activity has been linked to neuroinflammatory responses and excitotoxicity [50,138,139].

Indoles, another group of metabolites derived from tryptophan by gut bacteria, also play an important role in neuroimmune interactions. These compounds have been shown to influence the immune system by modulating inflammatory pathways and contributing to the homeostasis of the gut–brain axis [140]. Dysregulated indole production can promote neuroinflammation and has been implicated in the pathophysiology of neurodegenerative disorders by altering brain function and neuronal integrity [141].

Thus, the gut microbiota exerts a profound influence on the central nervous system by regulating neurotransmitter metabolism, immune responses, and neuroinflammatory pathways. Understanding these interactions offers potential therapeutic avenues for modulating the composition of the gut microbiota to attenuate the progression of mood disorders and neurodegenerative diseases.

### 2.5. Impact of Gut Dysbiosis on Neurodegenerative Diseases

The rising prevalence of neurodegenerative diseases, such as AD and PD, is closely linked to the increasing life expectancy of the world’s population [142,143]. As these diseases become more prevalent, the identification of mechanisms to slow or prevent their progression will be critical to reducing both the societal and healthcare burden. The term neurodegenerative diseases encompasses a wide range of disorders characterised by progressive loss of neuronal structure and function. These diseases are characterised by different pathological mechanisms and are generally grouped into major disorders, including AD, PD, Huntington’s disease, amyotrophic lateral sclerosis (ALS), and frontotemporal dementia [144,145].

Huntington’s disease is a genetic disorder caused by mutations in the HTT gene that lead to the accumulation of toxic huntingtin protein aggregates. The disease manifests as motor dysfunction, cognitive decline, and psychiatric disorders [146]. ALS, also known as Lou Gehrig’s disease, is a motor neuron disease characterised by progressive muscle weakness and paralysis due to the degeneration of motor neurons in the spinal cord and brain [147]. Frontotemporal dementia primarily affects the frontal and temporal lobes of the brain, resulting in profound changes in personality, behaviour, and language processing [148].

In addition to these major neurodegenerative diseases, other conditions contribute to neurodegeneration. Multiple sclerosis (MS) is an autoimmune disease in which the immune system attacks the myelin sheath of nerve cells, leading to demyelination, impaired signal transmission, and neurological dysfunction [149]. Spinocerebellar ataxias are a group of inherited disorders that cause degeneration of the cerebellum, resulting in impaired co-ordination, balance, and movement [150]. Prion diseases, such as Creutzfeldt-Jakob disease, result from the accumulation of misfolded prion proteins that induce neurotoxicity and lead to rapid brain degeneration [151].

A common pathological feature of these disorders is the aggregation of misfolded proteins, including β-amyloid and tau in AD [152], α-synuclein in Parkinson’s disease [153], and huntingtin in Huntington’s disease [154]. These aggregates contribute to oxidative stress, neuroinflammation, and mitochondrial dysfunction, exacerbating neuronal damage [155]. The interplay between protein misfolding, chronic inflammation, and impaired cellular homeostasis underscores the complexity of these diseases and highlights the need for targeted therapeutic interventions. Current research efforts focus on strategies to mitigate disease progression, including neuroprotective agents, gene therapy, and immunomodulatory approaches [156]. Understanding the molecular mechanisms underlying neurodegeneration is essential for the development of effective treatments aimed at preserving neuronal integrity and delaying the onset of debilitating symptoms.

### 2.6. Role of the Gut–Brain Axis, Dysbiosis, and Inflammation in Neurodegeneration

Shifts in gut microbiota can induce a chronic inflammatory state that contributes significantly to the development of neurodegenerative diseases, including AD and PD [122]. This imbalance can trigger an overactive immune response leading to increased production of pro-inflammatory cytokines, such as TNF-α, IL-6, and IL-1β. These cytokines can in turn activate microglial cells in the CNS, leading to neuroinflammation—a hallmark of neurodegenerative diseases [157]. In AD, chronic neuroinflammation exacerbates the accumulation of amyloid beta plaques and tau protein tangles, which disrupt neuronal function and accelerate cognitive decline [158]. Similarly, in Parkinson’s disease, inflammation is thought to contribute to the progressive loss of dopaminergic neurons in the substantia nigra, a critical feature of the disease pathophysiology [159]. The inflammatory response in the brain is closely linked to gut-derived microbial metabolites and immune signalling pathways, further highlighting the importance of the gut microbiota in modulating neuroinflammatory processes [55].

The role of inflammatory cytokines in neurodegeneration is well documented. Elevated levels of TNF-α, IL-6, IL-1β, and interferons are commonly found in both the blood and cerebrospinal fluid of patients with neurodegenerative diseases, suggesting a systemic inflammatory component [160]. Dysbiosis can exacerbate this inflammation by disrupting the integrity of the intestinal barrier, leading to increased intestinal permeability, often referred to as ‘leaky gut’ [161]. This allows bacterial endotoxins, such as LPS, to enter the systemic circulation, further amplifying inflammatory responses in the periphery and CNS [162].

The causal relationship between microbial disarray and gastrointestinal (GI) disorders remains a topic of ongoing debate. Such conditions as irritable bowel syndrome (IBS), functional dyspepsia, and IBD are often associated with microbial dysregulation, but it remains unclear whether dysbiosis is a consequence or a driver of chronic inflammation [163]. Changes in the microbial composition are observed in IBS and functional dyspepsia, but the precise mechanisms by which these changes contribute to such symptoms as abdominal pain, bloating, and altered bowel habits remain elusive [164]. In IBD, which is characterised by chronic inflammation of the gastrointestinal tract, the microbiota exhibits significant dysregulation, including reduced microbial diversity and increased prevalence of pro-inflammatory species, such as *Escherichia coli* and *Clostridium difficile*. However, whether these shifts initiate inflammatory processes or are secondary to the already dysregulated immune response remains uncertain [163].

This complexity highlights the need for further research to clarify whether impaired microbiome composition precedes or follows inflammation. Resolving this causality dilemma is crucial for the development of targeted therapies. If unbalanced gut flora is identified as the primary driver, such interventions as probiotics, prebiotics, dietary modification, and faecal microbiota transplantation (FMT) may be particularly effective. Conversely, if inflammation is the initiating factor, therapies aimed at immune modulation and restoration of epithelial barrier function should be prioritised [165].

C-reactive protein (CRP), a well-established biomarker of systemic inflammation, is elevated in several neurodegenerative diseases, including AD and PD [166]. Higher levels of CRP may indicate a chronic inflammatory state, possibly linked to an imbalance in the gut microbiota. Given the increasing recognition of gut-derived inflammation as a contributing factor to CNS disorders, CRP and other inflammatory markers may serve as valuable indicators of gut–brain axis dysfunction [167,168].

Thus, the interplay between impaired microbiome composition, systemic inflammation, and neurodegeneration highlights the gut–brain axis as a crucial factor in disease pathogenesis. Understanding the bidirectional relationship between microbial dysbiosis and immune activation will be essential for the development of novel therapeutic strategies aimed at modulating the gut microbiota, reducing neuroinflammation, and ultimately attenuating the progression of neurodegenerative diseases.

### 2.7. Role of Microbial Imbalance in Oxidative Stress and Inflammation

Gut dysbiosis has been shown to play a significant role in the pathogenesis of neurodegenerative diseases, such as AD and PD [122]. A healthy gut microbiota is essential for maintaining intestinal homeostasis, immune function, and metabolic processes, all of which have a direct impact on brain health [102]. However, disrupted gut microbial community leads to a decrease in microbial diversity and an overgrowth of harmful microorganisms that produce toxic metabolites. This imbalance disrupts the gut barrier and triggers systemic inflammation, which is a major contributor to neurodegeneration [165].

One of the main mechanisms by which microbial imbalance contributes to neurodegeneration is through oxidative stress. In normal conditions, beneficial gut bacteria produce SCFAs, such as butyrate, acetate, and propionate, which help to maintain the integrity of the gut barrier and reduce systemic oxidative stress [49,122]. However, in dysbiosis, the production of SCFAs is reduced, leading to increased permeability of the intestinal epithelium (“leaky gut”) and allowing the translocation of harmful bacterial metabolites, such as LPS [132]. LPS molecules activate immune cells and trigger the release of pro-inflammatory cytokines, including TNF-α, IL-6, and IL-1β, which can cross the BBB and exacerbate neuroinflammation [162]. Oxidative stress plays a key role in this process. ROS accumulate as a result of chronic inflammation, leading to neuronal damage and accelerating the progression of neurodegenerative diseases [169]. In AD, oxidative stress exacerbates the accumulation of amyloid β plaques and tau protein tangles [170], while in PD it contributes to the degeneration of dopaminergic neurons in the substantia nigra [171].

A considerable body of research has demonstrated that gut microbial dysregulation can result in the disruption of the intestinal epithelial barrier, thereby rendering it more permeable to potentially harmful substances [172]. In typical conditions, the intestinal barrier plays a crucial role in preventing the entry of noxious bacteria and toxins into the bloodstream. However, in the event of dysbiosis, pathogenic bacteria and their by-products, such as LPS, can traverse the compromised intestinal barrier and enter the circulatory system. These LPS molecules subsequently activate immune cells, thereby triggering a systemic inflammatory response [173,174]. Consequently, the release of pro-inflammatory cytokines and other inflammatory molecules into the bloodstream is initiated, ultimately reaching the brain where they activate glial cells and instigate neuroinflammation. This systemic inflammation directly exacerbates the progression of neurodegenerative diseases through aggravation of oxidative stress, damage to neurons, and impairment of cognitive and motor abilities [175].

**Figure 6:**
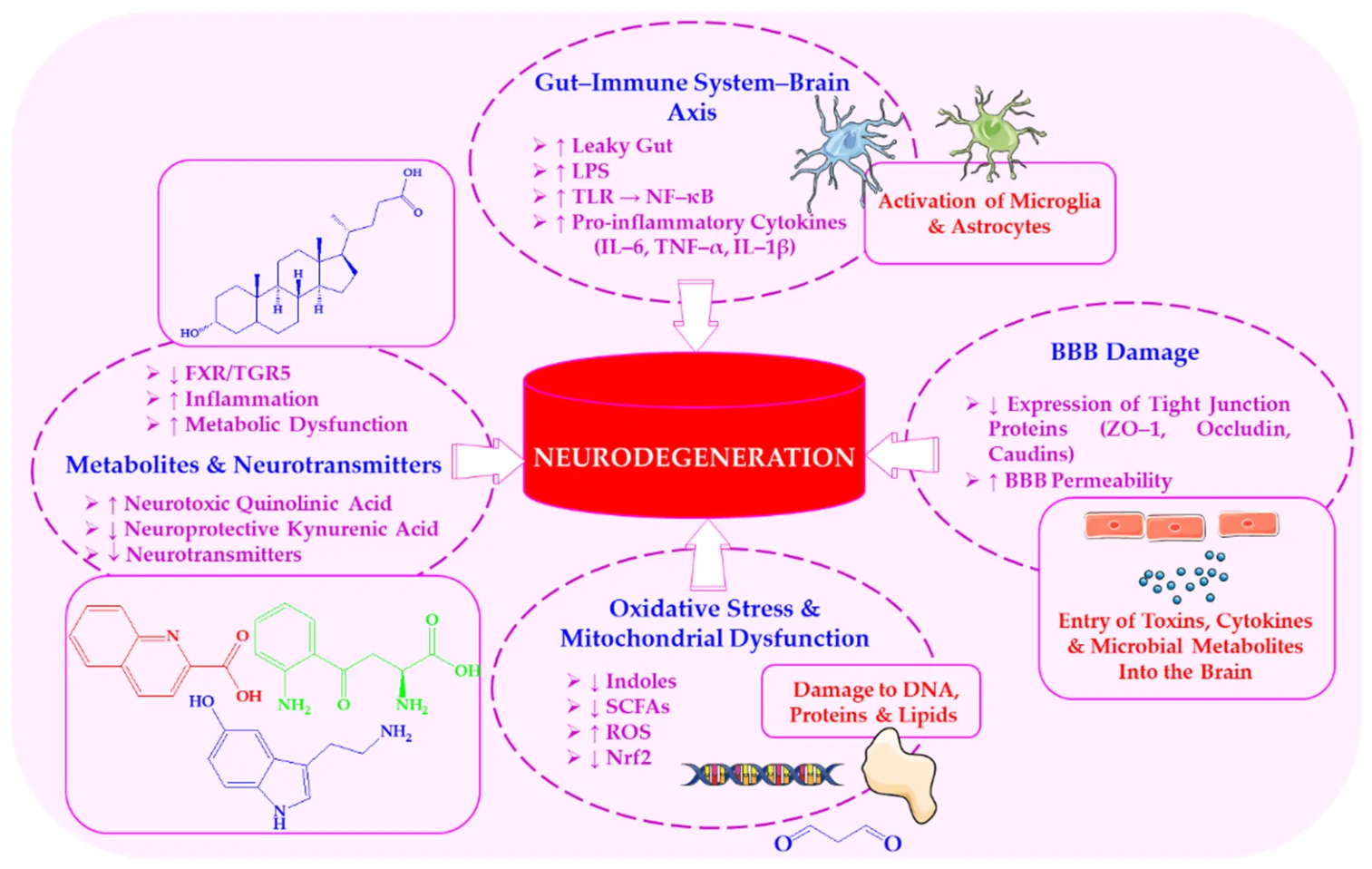
Molecular mechanisms linking gut microbiota to neurodegeneration. Gut microbiota influence neurodegeneration by promoting chronic inflammation, impairing the blood–brain barrier, altering neurotransmitters production, disrupting gut–brain axis communication, and contributing to oxidative stress and mitochondrial dysfunction. Image provided by Servier Medical Art (https://smart.servier.com), licensed under CC BY 4.0 (https://creativecommons.org/licenses/by/4.0/, accessed on 26 March 2026). Abbreviations: BBB—blood–brain barrier; DNA—deoxyribonucleic acid; FXR—farnesoid X receptor; IL-1β—interleukin-1 beta; IL-6—interleukin-6; LPS—lipopolysaccharides; NF-κB— nuclear factor kappa-light-chain-enhancer of activated B cells; Nrf2—nuclear factor erythroid 2-related factor 2; ROS—reactive oxygen species; SCFAs—short-chain fatty acids; TGR5—Takeda G-protein-coupled receptor 5; TLR—toll-like receptor; TNF-α—tumor necrosis factor alpha; ZO-1—zonula occludens-1; ↑—increase/upregulation; ↓—decrease/downregulation.

Emerging evidence suggests that shifts in gut microbiota disrupts the gut–brain axis through several interconnected pathways (Figure 6). Figure 6 illustrates the basic molecular mechanisms linking the gut microbiota to neurodegeneration. These include neurotransmitter production, regulation of the immune system, and modulation of VN signalling. The role of the gut microbiota in the production of neurotransmitters such as GABA and dopamine, which in turn regulate key physiological functions including motor activity, cognition and mood, has been a subject of considerable research in recent years [11]. Dysbiosis-induced alterations in neurotransmitter synthesis may contribute to neuropsychiatric symptoms often seen in neurodegenerative diseases [122]. In addition, disrupted gut microbial community can compromise the integrity of the BBB, making it more susceptible to the infiltration of neurotoxic molecules. The weakening of this protective barrier allows pro-inflammatory cytokines, ROS, and microbial metabolites to enter the CNS, further driving neurodegenerative processes [176].

Given the profound impact of altered gut microbiota on neurodegeneration, interventions aimed at restoring microbial balance have gained considerable interest. Probiotics and prebiotics have been shown to modulate the gut microbiota composition, enhance SCFA production, and reduce systemic inflammation [165,177]. Dietary modifications, such as increased fibre intake and consumption of polyphenol-rich foods, may also support gut health and reduce oxidative stress [178]. In addition, faecal microbiota transplantation is being explored as a potential therapeutic approach to restore a healthy microbiome and counteract neuroinflammatory processes [179].

Thus, the intricate relationship between gut disturbed microbial ecology, oxidative stress, and neuroinflammation underscores the critical role of the gut–brain axis in the pathogenesis of neurodegenerative diseases. Dysbiosis-induced microbial imbalance promotes systemic inflammation and disrupts gut–brain communication, accelerating neuronal damage and cognitive decline [177,180]. Strategies aimed at restoring gut microbiota homeostasis represent a promising avenue for mitigating the effects of neurodegeneration and improving patient outcomes [5]. Future research should continue to explore the potential of microbiome-targeted therapies in the prevention and treatment of neurodegenerative diseases.

### 2.8. Role of the Gut–Brain Axis in Parkinson’s Disease

**Figure 7:**
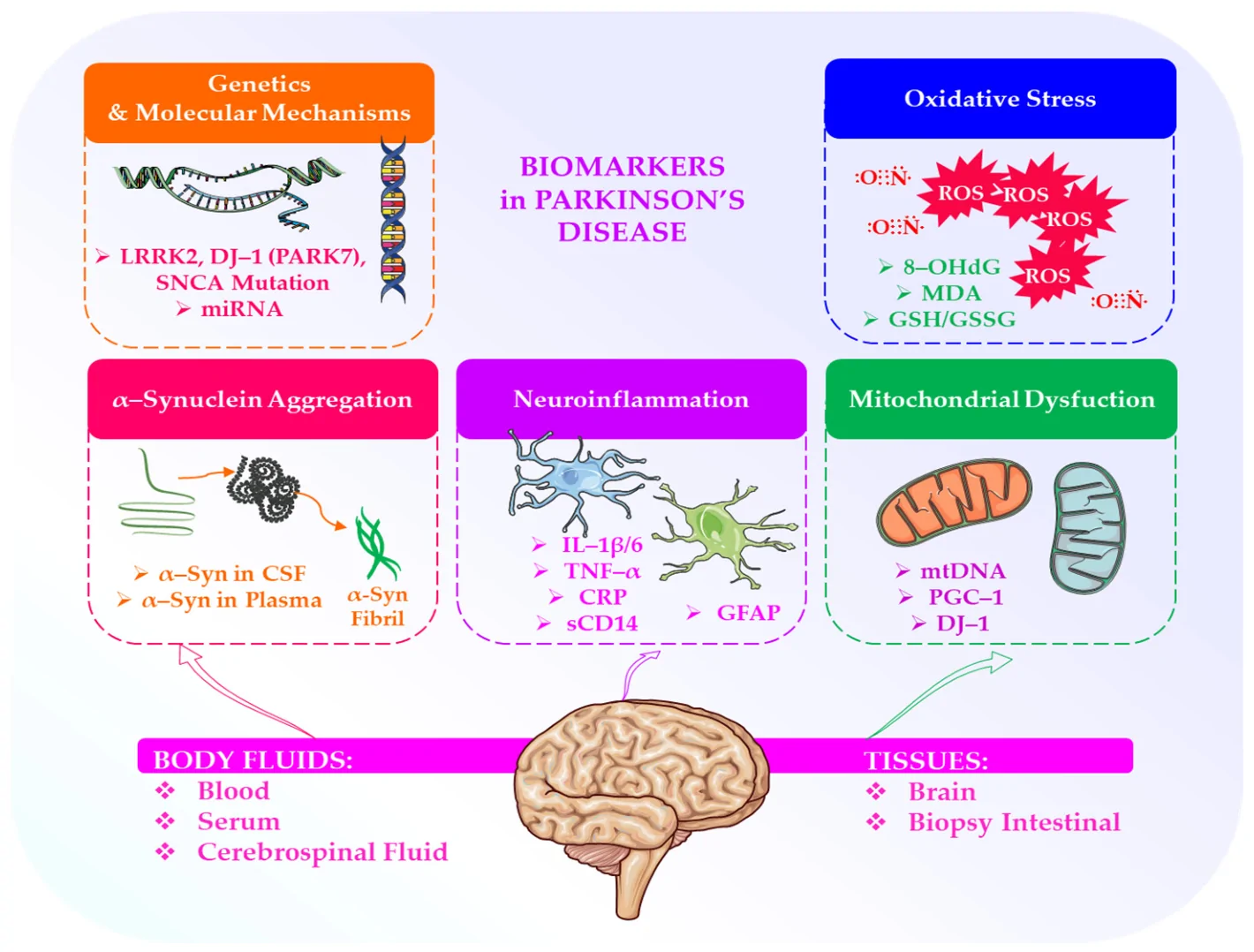
Biomarkers associated with molecular pathways in Parkinson’s disease. In Parkinson’s disease (PD), biomarkers are recognized as indicators of key molecular processes, including genetic and signaling pathways, α-synuclein aggregation, neuroinflammation, mitochondrial dysfunction, and oxidative stress. These interconnected mechanisms drive disease progression and are reflected by specific biomarkers that can be detected in body fluids and neural tissue. Image provided by Servier Medical Art (https://smart.servier.com), licensed under CC BY 4.0 (https://creativecommons.org/licenses/by/4.0/, accessed on 26 March 2026). Abbreviations: 8-OHdG—8-Hydroxy-2′-deoxyguanosine; α-Syn—alpha-synuclein; CRP—C-reactive protein; CSF—cerebrospinal fluid; DJ-1 (PARK7)—protein deglycase DJ-1; GSH—reduced glutathione; GSSG—oxidized glutathione; GFAP—glial fibrillary acidic protein; IL-1β—interleukin-1 beta; IL-6—interleukin-6; LRRK2—leucine-rich repeat kinase 2; MDA—malondialdehyde; mtDNA—mitochondrial DNA; NO—nitric oxide; PGC-1α—peroxisome; ROS—reactive oxygen species; sCD14—soluble CD14; SNCA—synuclein alpha; TNF-α—tumor necrosis factor alpha.

PD is a neurodegenerative disease that primarily affects dopaminergic neurons in the substantia nigra pars compacta. The pathological hallmark of PD is the accumulation of misfolded α-synuclein proteins that aggregate to form Lewy bodies. These aggregates are associated with neuronal dysfunction and cell death, contributing to the motor and non-motor symptoms observed in PD [181]. While the exact aetiology of PD remains unclear, genetic predisposition and environmental factors are known to contribute [182]. Figure 7 shows the most important biomarkers related to molecular pathways in Parkinson’s disease. Particularly in the early stages of the disease, these molecular changes trigger the activation of apoptotic pathways in neurons. Recently, the gut–brain axis has received increasing attention as a potential pathway involved in the pathogenesis of PD [183].

The loss of dopamine-producing neurons in the substantia nigra, a hallmark of PD, and the formation of Lewy bodies, which disrupt normal cellular function and contribute to neurodegeneration. The reduction in dopamine levels leads to motor symptoms such as tremor, rigidity and bradykinesia.

A key molecular feature of PD is neuroinflammation, which plays a crucial role in disease progression. Inflammatory processes are driven by the activation of microglia and astrocytes, leading to increased production of ROS and pro-inflammatory cytokines, such as IL-1 and TNF-α. These inflammatory mediators contribute to oxidative stress, mitochondrial dysfunction, and ultimately the loss of dopaminergic neurons [184]. Furthermore, oxidative damage is particularly prevalent in the substantia nigra due to the high metabolic activity of dopaminergic neurons and their susceptibility to mitochondrial dysfunction [27].

Emerging evidence suggests that alterations in the composition of the gut microbiota may play a critical role in the pathogenesis of PD [185]. Dysbiosis, defined as an imbalance in the gut microbial community, has been linked to the overproduction of pro-inflammatory cytokines, which can reach the CNS via the vagus nerve and exacerbate neuroinflammation [186]. In addition, microbial metabolites, such as SCFAs and LPS, have been implicated in neurodegenerative processes. SCFAs, including butyrate, propionate, and acetate, are essential for maintaining intestinal homeostasis and modulating immune responses [49]. However, alterations in SCFA production due to dysbiosis may contribute to increased intestinal permeability and systemic inflammation [132]. The presence of neurotoxic metabolites in PD patients suggests that gut microbiota dysfunction may be an early contributor to disease progression [185,187].

The VN plays a central role in bidirectional communication between the gut and the brain. It modulates the composition of the gut microbiota, regulates intestinal permeability, and transmits signals that influence neuroinflammatory responses [48]. Research has shown that the VN exerts anti-inflammatory effects by inhibiting inflammatory cell activity, which could potentially reduce neuroinflammation in Parkinson’s disease [78]. In addition, pathological studies have identified Lewy bodies in brainstem regions associated with VN nuclei in the early stages of PD, further supporting the hypothesis that PD may originate in the gut before spreading to the CNS [188,189].

The hypothesis that the VN serves as a critical mediator in PD has emerged from the evolving understanding of alternative pathways linking the gut microbiota and the CNS [190]. The gut–brain axis, modulated by the VN, is thought to play a central role in the onset and progression of PD, with dysfunction in this pathway potentially exacerbating neurodegenerative processes [183]. Research suggests that the VN is deeply involved in regulating the composition of the gut microbiota and the integrity of the gut wall, disruptions of which may contribute to the inflammation and motor dysfunction characteristic of PD [79]. The VN exerts its influence through direct innervation of smooth muscle cells and intramural ganglia within the gastrointestinal wall and through paracrine mechanisms involving the release of acetylcholine and other neuroactive modulators [191]. These processes are essential for maintaining intestinal homeostasis and immune regulation, both of which are compromised in PD [192]. Studies suggest that dysregulation of this pathway can lead to an imbalance in the gut microbiota, increased intestinal permeability, and inflammation, which may propagate neurodegeneration via gut–brain signalling [193]. In addition, activation of the VN has been shown to have anti-inflammatory effects, offering potential therapeutic applications in the treatment of PD by reducing inflammation in both the gut and CNS, thereby alleviating certain motor and non-motor symptoms of the disease [78,194,195].

Increased permeability in the presence of pre-existing pathology creates a vicious cycle in which microbial metabolites crossing the compromised barrier impair VN function, further exacerbating barrier dysfunction [112]. Evidence from some studies suggests that the influence of the gut microbiota extends beyond local effects, as microbial metabolites can be transmitted to the CNS via VN fibres [48]. This phenomenon has been demonstrated in Parkinson’s disease, where microbial metabolites interact with VN fibres and subsequently contribute to neurodegeneration [79]. A prevailing hypothesis is that neurotoxic metabolites entering the brain via the VN may serve as an initiating factor in the formation of Lewy bodies—aggregates of misfolded α-synuclein and other intracellular neuronal components characteristic of PD [196]. Indirect pathological support for this theory is provided by the observation that Lewy bodies appear in the brainstem in the early stages of the disease, particularly in regions containing VN nuclei [197,198]. However, even if this hypothesis is correct, the precise mechanisms underlying disease progression remain unclear, particularly in relation to the preferential degeneration of the substantia nigra.

Growing evidence implicates the gut microbiota in Parkinson’s disease (PD) pathogenesis through its role in neurotransmitter metabolism, immune modulation, and gut–brain communication. Altered microbial composition may impair dopamine and serotonin metabolism, contributing to both motor and non-motor symptoms of PD [65,185,199]. Dysbiosis is also linked to increased gut permeability and blood–brain barrier dysfunction, facilitating neurotoxic metabolite entry into the brain and promoting neuroinflammation [112,186,200]. Therapeutic strategies targeting the microbiota—such as probiotics, prebiotics, dietary interventions, and VN stimulation—have shown potential to restore microbial balance, reduce inflammation, and slow disease progression [78,185,186,194,195].

### 2.9. Role of the Gut Microbiome in Alzheimer’s Disease

**Figure 8:**
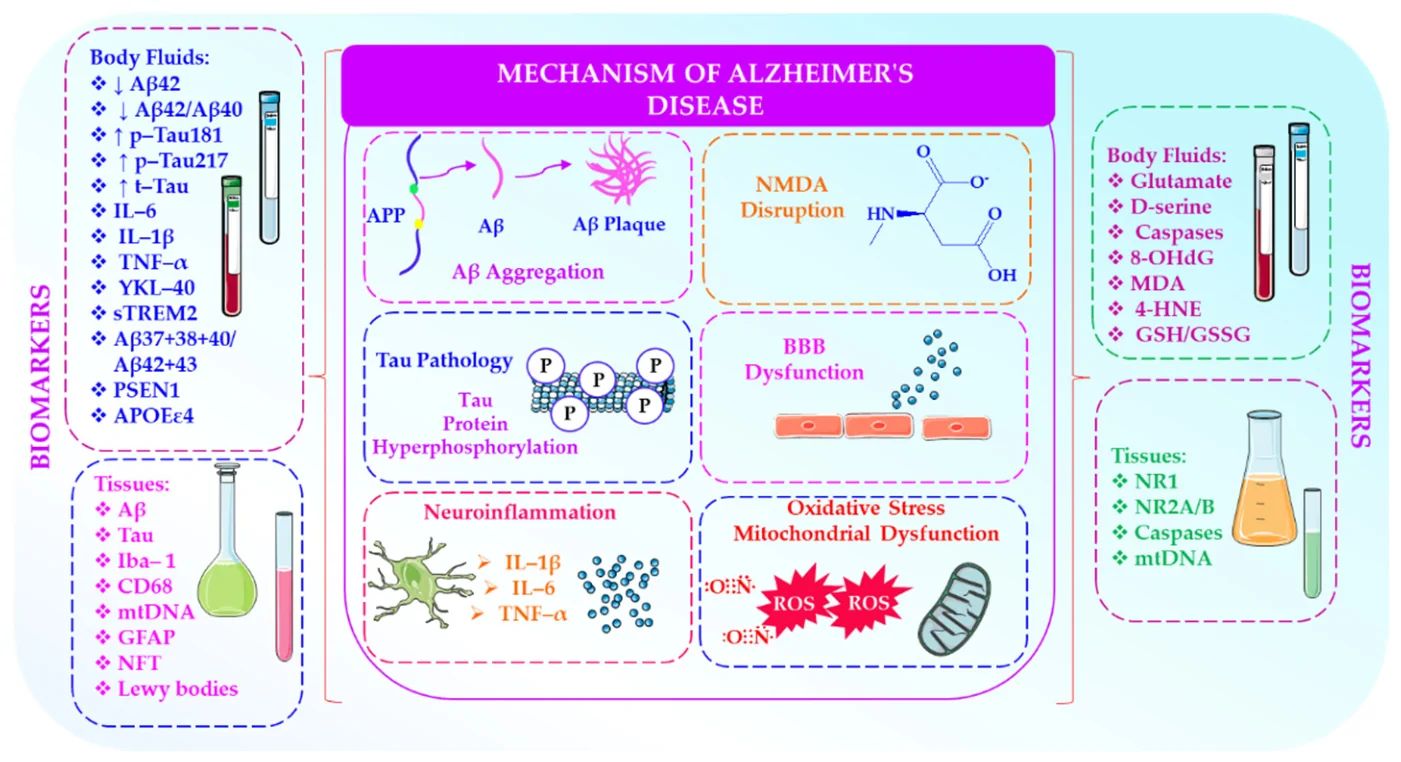
Mechanisms, biomarkers and molecular pathways of Alzheimer’s disease. Alzheimer’s disease is a multifactorial disorder characterized by amyloid-β (Aβ) accumulation, tau pathology, neuroinflammation, mitochondrial dysfunction, and vascular alterations. Increasing evidence highlights the role of the gut microbiota in modulating these processes, including Aβ aggregation, blood–brain barrier integrity, and oxidative stress. Associated biomarkers, such as inflammatory cytokines, tight junction proteins, and oxidative stress markers, can be detected in body fluids and tissues, providing valuable insights into disease mechanisms, diagnosis, and progression. Abbreviations: 4-HNE—4-hydroxynonenal; 8-OHdG—8-hydroxy-2′-deoxyguanosine; Aβ—amyloid-β; Aβ37+38+40/Aβ42+43—composite amyloid-β peptide ratios; Aβ42—amyloid-β 42; Aβ42/Aβ40—amyloid-β ratio; APOEε4—apolipoprotein E epsilon 4; APP—amyloid precursor protein; BBB—blood–brain barrier; CD68—a marker of activated microglia and macrophages; GFAP—glial fibrillary acidic protein; GSH—reduced glutathione; GSH/GSSG—glutathione ratio; GSSG—oxidized glutathione; Iba-1—ionized calcium-binding adapter molecule 1; IL-1β—interleukin-1 beta; IL-6—interleukin-6; MDA—malondialdehyde; mtDNA—mitochondrial DNA; NFT—neurofibrillary tangles; NMDA—N-methyl-D-aspartate receptor; NO—nitric oxide; NR1—NMDA receptor subunit NR1; NR2A/B—NMDA receptor subunits NR2A and NR2B; p-Tau181—phosphorylated Tau at threonine 181; p-Tau217—phosphorylated Tau at threonine 217; PSEN1—presenilin-1; ROS—reactive oxygen species; sTREM2—soluble triggering receptor expressed on myeloid cells 2; Tau—microtubule-associated protein Tau; TNF-α—tumor necrosis factor alpha; YKL-40—chitinase-3-like protein 1; ↑—increase/upregulation; ↓—decrease/downregulation.

AD is a neurodegenerative disorder that primarily affects memory and cognitive function. It is characterised by the accumulation of amyloid beta (Aβ) plaques and tau tangles in the brain, accompanied by neuroinflammation and neuronal cell death [201]. Recent research suggests that the gut microbiome may play a pivotal role in the development and progression of AD by influencing molecular mechanisms that contribute to disease pathology [202,203]. A growing body of evidence has identified several molecular biomarkers associated with AD that may be modulated by the gut microbiome, providing valuable insights into the pathophysiology of the disease [204]. The role of the gut microbiome in disease progression is gaining recognition through its involvement in molecular mechanisms, such as Aβ aggregation, neuroinflammation, disruption of BBB integrity, and mitochondrial dysfunction [98]. Gut-derived biomarkers, including inflammatory cytokines, tight junction proteins, and oxidative stress markers, may provide new insights into the underlying mechanisms of AD [205]. The mechanisms, biomarkers, and molecular pathways of AD are illustrated in Figure 8.

A hallmark of AD is the accumulation of Aβ peptides that form plaques in the brain [206]. Research has shown that the composition of the gut microbiome can influence both the production and clearance of Aβ [207]. The accumulation of Aβ plaques and tau tangles disrupts neuronal function, and dysbiosis has been implicated in modulating Aβ and tau metabolism either by influencing the immune response or by altering gut–brain signalling pathways involved in protein regulation [208]. A prevailing hypothesis is that specific gut bacterial species may regulate Aβ synthesis through the production of such metabolites as SCFAs, which have anti-inflammatory and antioxidant properties and may attenuate neurodegeneration [97,177]. Conversely, dysbiosis—characterised by an imbalance in the gut microbial composition—has been proposed to increase BBB permeability, thereby facilitating the accumulation and deposition of Aβ plaques [98]. In addition, elevated levels of gut-derived LPS and other neurotoxic metabolites have been shown to exacerbate amyloid pathology, highlighting their potential role as novel biomarkers of AD in the context of gut microbiome alterations [205,209].

Chronic neuroinflammation, driven primarily by microglial activation and subsequent release of pro-inflammatory cytokines, has been recognised as a key pathological feature of AD [160]. The influence of the gut microbiome on systemic inflammation is well established, with specific bacterial taxa modulating the secretion of inflammatory mediators [132,210]. In particular, the production of SCFAs, e.g., butyrate, by Firmicutes and Bacteroides has been shown to exert anti-inflammatory effects and contribute to neuroprotection against neuroinflammation [113]. However, dysbiosis, characterised by an overgrowth of pathogenic bacteria, can lead to increased levels of LPS, a known endotoxin capable of triggering a systemic inflammatory response [132]. The biomarkers of neuroinflammation in AD include elevated levels of TNF-α, IL-6, and IL-1β cytokines, which may indicate a microbial imbalance [211]. Furthermore, elevated serum levels of C-reactive protein have been associated with both neuroinflammation and gut dysbiosis in AD patients, suggesting a potential mechanistic link between the gut microbiome and neuroinflammatory pathways [180].

Research has highlighted the critical role of BBB integrity in protecting the brain from neurotoxic substances. The gut microbiome has the potential to modulate BBB permeability through the release of metabolites, such as SCFAs, which play a key role in maintaining tight junction integrity [112,212]. Microbiota disruption, characterised by an overgrowth of pathogenic bacteria, can lead to increased production of gut-derived toxins, including LPS, which can enter the bloodstream and initiate systemic inflammation [132]. This inflammatory response can subsequently compromise the integrity of the BBB, facilitating the entry of harmful substances, such as amyloid-beta peptides, pro-inflammatory cytokines, and toxins into the brain [213]. Biomarkers of BBB dysfunction in AD have been identified, including serum levels of tight junction proteins, such as claudins and occludins [214]. Changes in the expression of these proteins, influenced by gut-derived metabolites, may reflect the degree of BBB dysfunction in AD patients [215].

Studies have highlighted the central role of mitochondrial dysfunction in the pathogenesis of AD, resulting in impaired cellular energy production and increased oxidative stress [216]. The gut microbiome has been identified as a source of metabolites, such as ammonia and butyrate, which affect mitochondrial function [217]. Dysbiosis, defined as an imbalance in the composition of the microbiome, can lead to the overproduction of harmful substances, e.g., ammonia, which can accumulate in the brain and disrupt mitochondrial activity [218,219]. This mitochondrial dysfunction in turn contributes to increased oxidative stress, exacerbating neuronal damage [220]. Biomarkers of mitochondrial dysfunction in AD include decreased mitochondrial DNA, decreased ATP production, and increased levels of ROS [221]. In addition, markers of oxidative damage, such as 8-hydroxy-2′-deoxyguanosine and malondialdehyde, indicate oxidative injury in neurons and may be associated with changes in the gut microbiome composition [222].

The relationship between AD and the gut microbiome has been studied extensively. Increased levels of microbial nitric oxide (NO) have been found to exacerbate oxidative stress, particularly in cells with limited resistance, such as cortical neurons [223]. Cortical neurons are particularly vulnerable to hypoxia and oxidative stress due to their relatively weak antioxidant defences and high metabolic demands [224]. Consequently, these effects primarily affect cortical neurons. The role of SCFAs in this process is also significant, as they have been shown to exert neuroprotective effects on both cortical and hippocampal neurons. The proposed mechanism involves a reduction in mitochondrial ROS production and an increase in antioxidant defences [49,225].

The neuroprotective effect of SCFAs is thought to be critical in mitigating oxidative damage to neurons in brain regions that are particularly vulnerable in neurodegenerative diseases, such as AD and PD [177,226]. SCFAs are thought to regulate mitochondrial function and activate endogenous antioxidant pathways, playing a key role in protecting neurons from oxidative stress-induced damage [113,130]. Furthermore, modulation of the gut microbiome represents a promising therapeutic strategy to control the progression of these neurodegenerative conditions, highlighting the complex relationship between gut-derived metabolites and brain health [5].

Lukiw et al. (2018) conducted a study investigating the effect of gut microbiome-derived LPS on the expression of the light chain of NF-L gene in nerve tissue. The researchers observed that LPS inhibited the expression of this gene, with a corresponding decrease in its mRNA and NF-L levels, which are indicative of degenerative changes in the brain. Such changes have been linked to neuronal and synaptic atrophy, a hallmark of AD [131]. The importance of NF-L in neuronal function and structural integrity is well established, and alterations in its expression are thought to be central to neurodegeneration [227]. In AD, reduced NF-L expression serves as a marker of neuronal damage and loss. The study conducted by Lukiw et al. (2018) highlights two key findings: first, the pathogenesis of neurodegenerative diseases, including AD, is associated with genetic changes, specifically the transcriptional regulation of specific genes, and second, metabolites from the human microbiome can directly influence gene expression in neural tissue. These findings suggest a direct molecular pathway by which gut-derived microbial products, such as LPS, may affect brain function and contribute to the progression of AD disease [131].

Numerous studies have investigated the relationship between trimethylamine N-oxide (TMAO) and the development of AD [228,229]. TMAO is formed during the catabolism of exogenous lipids by the gut microbiota and has been implicated in the pathogenesis of several diseases, including atherosclerosis and cardiovascular disease [230]. Elevated levels of TMAO in cerebrospinal fluid (CSF) have been observed in individuals with dementia associated with AD, compared to those without cognitive impairment [231]. Furthermore, elevated levels of TMAO have been associated with key biomarkers of AD, such as phosphorylated tau and NF-L [232]. Although the precise role of hyperphosphorylated tau in AD remains uncertain, it is thought to contribute to cytoskeletal disruption [233]. In addition, TMAO has been shown to divert amyloid precursor protein (APP) metabolism towards the amyloidogenic pathway, increasing β-secretase expression and β-amyloid production, although the underlying mechanism remains unclear [234]. Elevated TMAO levels are also associated with nutritional problems and increased intestinal barrier permeability, conditions that may exacerbate the effects of other microbial factors [230].

Thus, there is growing evidence linking the gut microbiome to the pathogenesis of AD through multiple mechanisms, including Aβ aggregation, neuroinflammation, BBB disruption, mitochondrial dysfunction, and oxidative stress. Gut-derived metabolites, such as SCFAs, TMAO, and LPS, appear to influence neurodegenerative pathways, highlighting the gut–brain axis as a critical factor in AD. It can be concluded that the gut microbiome plays a critical role in the development of AD through several mechanisms, e.g., the production of such metabolites as TMAO and SCFAs [235], their effects on neuroinflammation, oxidative stress, and neuronal health [180], and changes in gene expression induced by microbial metabolites that subsequently influence neuronal components, such as NF-L [205]. These findings suggest a link between changes in the gut microbiome and the molecular mechanisms that drive the development and progression of AD. Identifying gut-derived biomarkers and understanding their role in AD progression may provide novel diagnostic and therapeutic strategies. Modulation of the gut microbiome through probiotics, dietary interventions, and targeted microbiome therapies may represent promising avenues to mitigate AD-related neurodegeneration and improve patient outcomes.

### 2.10. Role of the Gut Microbiome in the Pathogenesis and Progression of Amyotrophic Lateral Sclerosis (ALS)

ALS is a prototypical neurodegenerative disease characterised by progressive degeneration and subsequent death of motor neurons in both the brain and spinal cord. This progressive loss of motor function is ultimately fatal, often leading to complete paralysis and death within a few years of diagnosis [236]. The molecular mechanisms underlying ALS have been shown to involve several pathological pathways, including impaired protein homeostasis, oxidative stress, and the accumulation of toxic protein aggregates [237]. Peggion et al. (2022) identified a key process in the pathogenesis of ALS—a mutation of a gene encoding superoxide dismutase 1 (*SOD1*) resulting in the production of misfolded proteins [238]. This misfolded protein accumulates in neurons, inducing cellular toxicity and contributing to oxidative damage. In addition to *SOD1*, mutations in other genes, such as *TDP-43* and *FUS*, have been implicated in ALS [239]. These mutations lead to abnormal protein folding and disrupt cellular processes, such as nucleocytoplasmic transport and RNA metabolism, further exacerbating neuronal dysfunction [240].

Another critical mechanism contributing to ALS is mitochondrial dysfunction [241]. Mitochondria are central to cellular energy production, and their impairment leads to increased oxidative stress and loss of energy homeostasis in motor neurons. Damaged mitochondria can also trigger cell death programmes, such as apoptosis and necrosis, further exacerbating neuronal dysfunction [242]. Loss of mitochondrial integrity has been observed in both sporadic and familial ALS, providing a strong link between mitochondrial dysfunction and disease progression [241]. In addition, activated microglia, the resident immune cells of the CNS, play a key role in the development of ALS. Their overactivation leads to chronic neuroinflammation, which in turn exacerbates neuronal injury and accelerates disease progression [243]. Microglia are involved in maintaining homeostasis in the CNS, but their prolonged activation in ALS can produce an inflammatory environment that worsens neuronal survival [54]. Consequently, the interplay of these molecular mechanisms—protein misfolding, mitochondrial dysfunction, and neuroinflammation—culminates in progressive motor neuron degeneration, leading to loss of voluntary muscle control and ultimately death [244].

In recent years, the link between ALS and the gut microbiome has gained increasing attention in the research community [245]. Although the precise mechanisms underlying this relationship are not yet fully understood, there is a growing body of evidence suggesting that the gut microbiome may play a role in the development and progression of ALS. A prevailing hypothesis is that the gut microbiome may modulate immune responses and inflammatory processes, which are thought to be integral to the pathogenesis of ALS [246,247]. Research has shown that changes in the composition of the microbiome can affect the activation of microglia, the immune cells of the CNS, which contribute to neuroinflammatory processes in the brain and spinal cord [120]. Excessive microglial activation, which accelerates neuronal degeneration, is often seen in ALS patients, both in preclinical models and in human studies [248,249,250]. It has been suggested that an imbalanced microbiome may also affect the levels of dopamine and serotonin neurotransmitters, which play a key role in regulating nervous system function and may influence the course of the disease [12]. Dysbiosis, or an imbalance in the gut microbiota, could potentially affect the regulation of these neurotransmitters, adding another layer to its contribution to the ALS pathology [251,252].

There is also evidence that the microbiome influences protein metabolism in the body, particularly in relation to mutations in such genes as *SOD1* and *TDP-43*, which are associated with ALS [247]. Some studies have suggested that gut bacteria may influence the folding and aggregation of misfolded proteins, directly affecting the pathogenic pathways that lead to motor neuron degeneration [252]. In addition, changes in the microbiome may lead to perturbations in the oxidative stress response, a critical mechanism underlying cellular damage in ALS [247,253]. Reduced diversity in the microbiome, particularly in bacteria responsible for short-chain fatty acid metabolism, may increase oxidative stress and inflammatory states in the body, further exacerbating disease progression [246,247].

Therefore, modulation of the microbiome through diet, probiotics, or prebiotics is emerging as a promising avenue for adjunctive therapies in the treatment of ALS [252,254]. Although further studies are needed to clarify the precise mechanisms by which the microbiome influences ALS, these preliminary findings suggest that the gut microbiome may play a significant role in the onset and progression of ALS [245,246,247]. The gut–brain axis, a bidirectional communication pathway between the gastrointestinal tract and the CNS, has opened up new avenues of research that may yield novel therapeutic strategies [8]. The elucidation of the impact of the gut microbiota composition on neuroinflammation, mitochondrial function, and protein metabolism in ALS could provide invaluable insights into potential treatment options.

### 2.11. Possible Role of Antioxidants in Gut–Brain Axis Homeostasis and Treatment of Neurodegenerative Diseases

Oxidative stress is a key pathological factor in neurodegenerative diseases, contributing to neuronal damage through the overproduction of ROS and an imbalance in antioxidant defence mechanisms [223]. In normal physiological conditions, ROS are produced as a natural by-product of cellular metabolism, particularly within mitochondria, where they play essential roles in signalling and cellular homeostasis. However, in pathological conditions, ROS levels increase significantly, overwhelming the body’s antioxidant defences and leading to cellular damage [16]. This damage manifests itself in a variety of ways, including lipid peroxidation, protein aggregation, and DNA mutations, all of which contribute to the progression of neurodegenerative diseases, such as AD and PD [20].

In AD, oxidative stress plays a central role in accelerating the pathological hallmarks of the disease, including Aβ plaque formation and tau protein hyperphosphorylation [170]. The accumulation of Aβ plaques is thought to result from the aggregation of misfolded proteins, driven in part by oxidative damage to proteins and lipids in the brain. This aggregation contributes to neuronal damage and disrupts synaptic function, ultimately leading to cognitive decline [255]. In addition, oxidative stress triggers tau hyperphosphorylation, which leads to the formation of neurofibrillary tangles. These tangles further impair neuronal communication and contribute to the overall neurodegeneration observed in AD patients [256]. The exacerbation of oxidative stress has also been shown to impair the BBB, potentially facilitating the entry of peripheral immune cells into the brain, which may further amplify neuroinflammation in AD [257].

Similarly, oxidative stress is a critical aspect of the deterioration of dopaminergic neurons in the substantia nigra, an indispensable region of the brain involved in motor control. The death of these neurons leads to a decrease in dopamine levels, which contributes to the manifestation of the motor symptoms characteristic of Parkinson’s disease, including tremor, rigidity, and bradykinesia [14]. Oxidative damage in dopaminergic neurons leads to mitochondrial dysfunction, exacerbating ROS production and creating a vicious cycle of oxidative damage [258]. In addition, the accumulation of misfolded proteins, including α-synuclein, is a hallmark of Parkinson’s disease pathology [153]. Oxidative stress promotes the formation of Lewy bodies, which consist of aggregated α-synuclein and further impair cellular function, leading to neuronal death and exacerbating motor dysfunction [181].

Another critical feature shared by AD and PD is the mitochondrial dysfunction associated with oxidative stress [259]. Mitochondria are the cell’s main source of energy, and elevated levels of ROS cause damage to mitochondrial components, including lipids, proteins, and DNA. This results in mitochondrial dysfunction, leading to further ROS production and a decrease in cellular energy availability [128]. This dysfunction is particularly detrimental in neurons due to their high energy requirements, leading to neuronal death and contributing to the progressive nature of neurodegenerative diseases [260]. Cognitive decline in AD and motor dysfunction in PD have been linked to chronic neuroinflammation, emphasising the therapeutic potential of targeting oxidative stress and inflammatory pathways [261,262]. The inflammation in question is driven by microglial activation and the release of pro-inflammatory cytokines in response to oxidative damage, which in turn disrupts neuronal function and synaptic integrity [175,223]. Mitochondrial dysfunction is a key upstream contributor to this cascade, as it is able to impair calcium homeostasis and exacerbate oxidative stress and neuroinflammatory processes [261].

Understanding the contribution of oxidative stress to the molecular mechanisms of AD and PD provides critical insights into potential therapeutic interventions. The development of effective therapeutic interventions that target oxidative stress and its downstream effects, such as protein aggregation, mitochondrial dysfunction, and neuroinflammation, holds great promise for delaying the onset or slowing the progression of these devastating diseases [263]. Such interventions could effectively modulate oxidative stress, improve mitochondrial function, and reduce neuroinflammation [264]. Therefore, the identification of antioxidants or small molecules that can modulate oxidative stress remains of great importance [263]. In addition, personalised therapeutic approaches that take into account the individual oxidative stress profile of patients could improve the efficacy of these treatments.

Antioxidants, including polyphenols, vitamins, and flavonoids, have been shown to play an important role in modulating the gut microbiota to reduce oxidative stress [37,265]. One of the main mechanisms by which antioxidants exert their beneficial effects is by promoting the growth of beneficial gut bacteria, such as *Bifidobacterium* and *Lactobacillus* [178]. These bacteria are known for their ability to ferment dietary fibre and produce beneficial metabolites, particularly SCFAs, including acetate, propionate, and butyrate [266]. SCFAs are critical for maintaining gut health by reducing oxidative stress and inflammation in the gut [267]. In particular, SCFAs have been shown to reduce the production of ROS in intestinal cells, thereby protecting intestinal epithelial cells from oxidative damage and helping to maintain a healthy intestinal mucosa [268].

In addition to promoting the growth of beneficial bacteria, antioxidants also influence the microbial composition by suppressing the growth of pathogenic microbes. For example, polyphenols and flavonoids can directly inhibit the growth of harmful bacteria, such as *Escherichia coli* and *Clostridium* species, which can induce intestinal inflammation and exacerbate oxidative stress [178]. By restoring a balanced gut microbiota, antioxidants help reduce the overproduction of harmful metabolites and inflammatory cytokines, thereby alleviating oxidative stress and maintaining intestinal homeostasis [269]. This modulation of microbial diversity is critical for overall health, as dysbiosis has been implicated in numerous chronic diseases, including inflammatory bowel disease and neurodegenerative conditions [270,271].

In addition, antioxidant molecules can influence the gut microbiota by modulating gene expression in both microbial and host cells [37]. A key pathway in this process is the activation of the Nrf2 pathway in intestinal epithelial cells [272]. The Nrf2 pathway is a key regulator of the cellular antioxidant response, which helps to protect against oxidative damage by increasing the expression of antioxidant enzymes, such as superoxide dismutase and glutathione peroxidase [273,274]. Antioxidants have been shown to reduce ROS levels in the gut, thereby creating a less oxidative environment that promotes a healthier gut microbiota and supports the integrity of the gut barrier [265,275].

In addition to their local effects in the gut, antioxidants play a crucial role in the bidirectional communication between the gastrointestinal system and the CNS [49]. One of the key signalling molecules in this process is SCFAs, which are produced by gut bacteria and enter the bloodstream. SCFAs have been shown to reduce inflammation and oxidative stress in the gastrointestinal tract. They can also modulate neuroinflammation in the brain, thereby exerting a neuroprotective effect [113]. By supporting a healthy gut microbiota and promoting SCFA production, antioxidants help regulate inflammatory signals, thereby protecting neurons from oxidative damage and contributing to neuronal health [36]. This highlights the potential of antioxidants as a therapeutic strategy for neurodegenerative diseases associated with both gut microbial disarray and oxidative stress [38].

The process of gut–brain communication, facilitated by antioxidants and their effects on the microbiota, involves several molecular signalling pathways. These include the immune response in the gut and activation of the VN [276]. The ability of antioxidants to modulate the production of pro-inflammatory cytokines is important [277]. In addition, antioxidants help regulate the activity of immune cells in the gut, preventing systemic inflammation from adversely affecting brain function and exacerbating oxidative stress within neuronal cells [265].

In conclusion, the emerging role of antioxidants in modulating the gut microbiota represents an exciting avenue for the development of novel therapeutic strategies. By influencing both local gut health and systemic inflammatory responses, antioxidants may offer a promising approach to mitigate oxidative stress and associated diseases, including neurodegenerative disorders [37,265]. Further research is needed to unravel the complex interactions between antioxidants, the gut microbiota, and the CNS and to explore their potential in clinical applications to improve health and prevent disease.

### 2.12. Role of Antioxidants in Modulating Molecular Pathways to Protect Neuronal Health in Neurodegenerative Diseases

Antioxidants have been shown to play a crucial role in regulating molecular pathways that provide protection against oxidative stress, a major contributor to neurodegenerative diseases, such as AD and PD [17]. Oxidative stress results from an imbalance between the production of ROS and the body’s ability to neutralise them through antioxidant defences [16]. In neurodegenerative diseases, oxidative stress leads to cell damage, inflammation, and neuronal death, accelerating disease progression [20]. As a master regulator of the cellular antioxidant response, Nrf2 controls the expression of key antioxidant enzymes such as glutathione peroxidase and superoxide dismutase [273,274], and its activation represents one of the principal mechanisms through which antioxidants exert their neuroprotective effects. These enzymes neutralise ROS and reduce oxidative damage to neuronal cells, thus playing a key role in maintaining redox balance [16]. In the context of neurodegenerative diseases, the activation of the Nrf2 pathway is crucial to support neuronal survival, prevent oxidative damage, and mitigate disease progression [278].

Another critical pathway modulated by antioxidants is the NF-κB pathway, which plays a central role in inflammation by regulating the expression of pro-inflammatory cytokines, such as TNF-α and IL-6 [277]. Chronic inflammation is a hallmark of neurodegenerative diseases, including AD and PD, where over-activation of NF-κB leads to neuronal damage and exacerbates oxidative stress [279,280]. Antioxidants, particularly polyphenols and flavonoids, have been shown to inhibit NF-κB activation, leading to a reduction in pro-inflammatory cytokine levels [281]. This inhibition not only reduces oxidative stress but also helps to protect neurons from inflammatory damage, highlighting the dual role of antioxidants in managing both oxidative and inflammatory pathways in the neurodegenerative environment [17].

**Figure 9:**
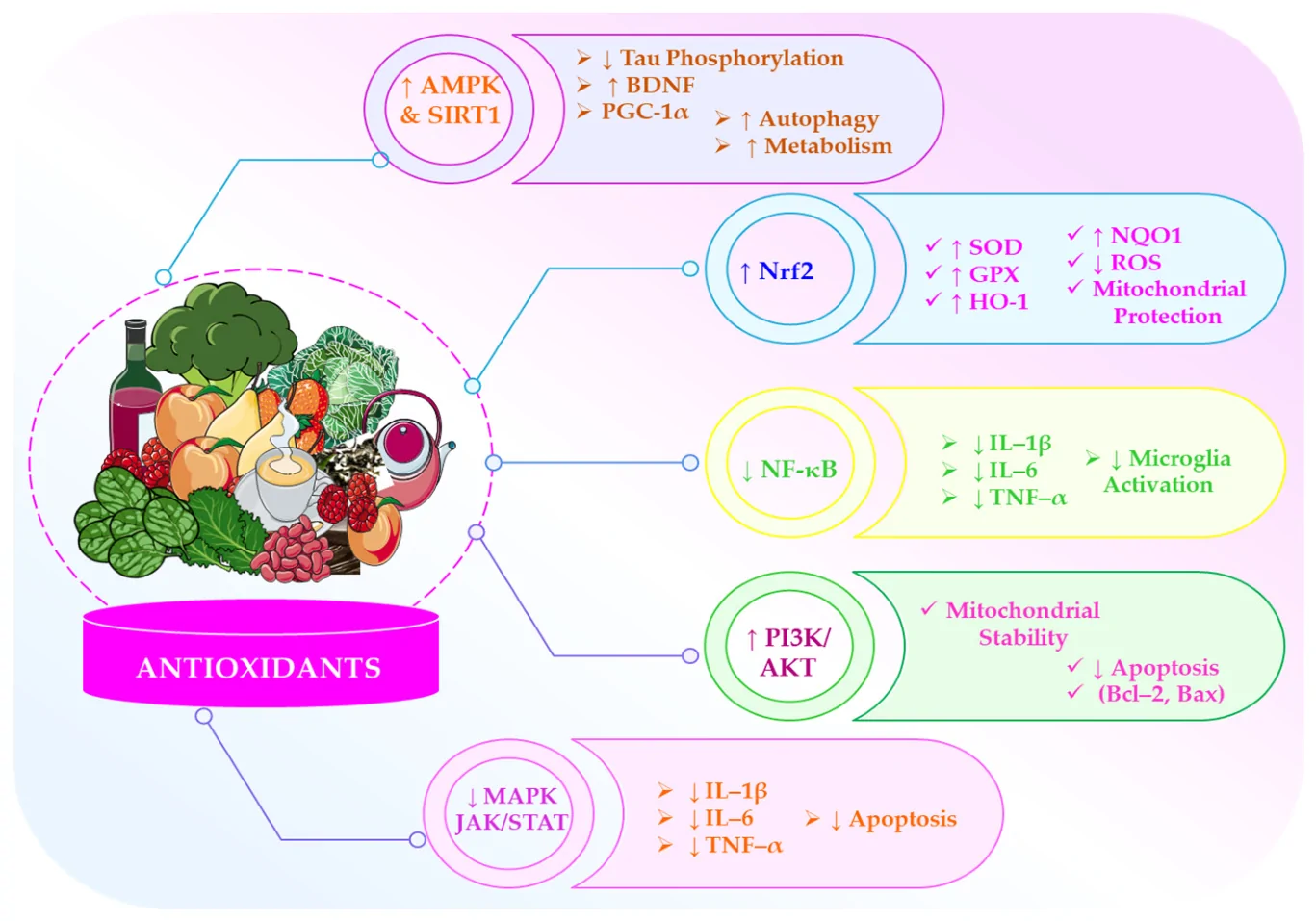
Antioxidant-mediated modulation of molecular pathways in neurodegenerative diseases. Molecular mechanisms and signaling pathways illustrate how antioxidants influence key processes involved in neurodegenerative diseases, providing protection against oxidative stress, neuroinflammation, and cellular damage. Antioxidants can modulate mitochondrial function, reduce reactive oxygen species production, and regulate inflammatory signaling pathways, thereby preserving neuronal integrity and function. Abbreviations: AKT—protein kinase B; AMPK—AMP-activated protein kinase; Bax—Bcl-2-associated X protein; BDNF—brain-derived neurotrophic factor; Bcl-2—B-cell lymphoma 2; GPX—glutathione peroxidase; HO-1—heme oxygenase-1; IL-1β—interleukin-1 beta; IL-6—interleukin-6; JAK—Janus kinase; MAPK—mitogen-activated protein kinase; NF-κB —nuclear factor kappa-light-chain-enhancer of activated B cells; NQO1—NAD(P)H quinone dehydrogenase 1; Nrf2—nuclear factor erythroid 2-related factor 2; PGC-1α—peroxisome; PI3K—phosphoinositide 3-kinase; ROS—reactive oxygen species; SIRT1—sirtuin 1; SOD—superoxide dismutase; STAT—signal transducer and activator of transcription; Tau—microtubule-associated protein Tau; TNF-α—tumor necrosis factor alpha; ↑—increase/upregulation; ↓—decrease/downregulation.

In addition to Nrf2 and NF-κB, antioxidants also affect the phosphoinositide 3-kinase (PI3K)/AKT pathway, an important molecular mechanism that promotes cell survival and regulates metabolic processes critical for neuronal health [282]. When activated, this pathway supports neuronal survival, inhibits apoptosis, and promotes cellular repair mechanisms. Antioxidants have been shown to enhance the PI3K/AKT pathway, leading to the activation of downstream targets that further suppress apoptosis and promote neuronal resilience [282]. By reducing ROS production and stabilising mitochondrial function, antioxidants ensure that neurons maintain proper energy production and cellular integrity [283]. The PI3K/AKT pathway is particularly beneficial in Parkinson’s disease, where dopaminergic neurons are highly susceptible to oxidative damage and mitochondrial dysfunction [284]. As shown in Figure 9, antioxidants can affect several molecular mechanisms and pathways underlying neurodegenerative diseases, providing protection against oxidative stress, inflammation, and cellular damage.

In addition, antioxidants modulate the mitogen-activated protein kinase (MAPK) pathway, which plays an important role in neuronal protection [36]. The MAPK pathway consists of several sub-pathways, including the extracellular signal-regulated kinase (ERK) and c-Jun N-terminal kinase (JNK) pathways, which regulate cellular responses to stress [285]. Dysregulation of MAPK signalling has been implicated in the pathogenesis of neurodegenerative diseases, leading to increased ROS production and neuronal apoptosis [286]. Antioxidants help to balance MAPK activity by suppressing the damaging JNK pathway and promoting the protective ERK pathway [287]. This modulation has been shown to reduce oxidative damage and support neuroprotection in such diseases as AD, where MAPK dysregulation contributes to neuronal injury [288].

In summary, antioxidants influence the interplay between these critical molecular pathways, creating a synergistic effect that enhances their neuroprotective properties. For example, the interaction between the Nrf2 and NF-κB pathways ensures a coordinated response to oxidative and inflammatory stress [289]. In addition, the activation of the PI3K/AKT pathway supports Nrf2 function by enhancing the production of antioxidant enzymes, providing further protection against oxidative damage. By modulating these interrelated pathways, antioxidants not only reduce ROS and inflammation but also promote neuronal survival, repair, and overall brain health [290]. This multifaceted molecular mechanism highlights the therapeutic potential of antioxidants in targeting the molecular basis of neurodegenerative diseases [38]. As research in this area progresses, antioxidants may emerge as key therapeutic agents in the management and treatment of such disorders as AD, PD, and other neurodegenerative diseases.

The growing understanding of the role of the gut microbiota in neurodegenerative diseases is opening up new avenues for therapeutic intervention. Recent studies have highlighted the intricate connection between the gut and the brain, often referred to as the “gut–brain axis”, suggesting that maintaining a healthy gut microbiota may offer new strategies for managing or even preventing neurodegenerative diseases [18,186]. Strategies such as the use of probiotics, prebiotics, and dietary changes are emerging as potential means of restoring the balance of the gut microbiota, which could help reduce neuroinflammation and improve brain function [291]. Probiotics, which introduce beneficial bacteria into the gut, have been shown to reduce systemic inflammation and improve gut–brain communication [292]. Prebiotics, which act as a food source for beneficial gut bacteria, can help increase the growth of these bacteria, further improving the gut–brain connection and potentially reducing neuroinflammation [291]. In addition, dietary changes, such as increasing fibre intake, adopting a Mediterranean diet rich in antioxidants, and including omega-3 fatty acids, can positively influence the composition of the gut microbiota and reduce the risk or progression of neurodegenerative diseases [293].

Probiotics exert a range of beneficial effects through both immunological and non-immunological mechanisms. Immunologically, they activate local macrophages, stimulate immunoglobulin A (IgA) production both locally and systemically, and improve mucosal immunity [294]. Probiotics also modulate cytokine responses to promote a balanced immune response, which helps to induce tolerance to dietary antigens, thereby minimising hypersensitivity reactions [295]. On the non-immunological front, probiotics participate in digestion and compete with pathogenic bacteria for nutrients [296]. They alter local pH levels to create a hostile environment for pathogens, produce bacteriocins to inhibit harmful bacteria, and scavenge superoxide radicals to reduce oxidative stress [297]. In addition, probiotics stimulate the production of epithelial mucin, which strengthens mucosal defences, improves the integrity of the intestinal barrier, and reduces intestinal permeability [298]. They also compete with pathogens for adhesion receptors, limiting pathogen colonisation, and modify pathogen-produced toxins to mitigate their harmful effects [294,299]. These combined actions underscore the critical role of probiotics in supporting gut health and overall systemic well-being [298].

Probiotics can be incorporated into a variety of foods, pharmaceutical preparations, and dietary supplements. The most commonly used probiotics include species of *Lactobacillus* and *Bifidobacterium* as well as the yeast *Saccharomyces boulardii* and certain strains of *Escherichia coli*, *Bacillus*, and *Clostridium butyricum* [300]. Probiotic formulations may also contain live cultures of *Streptococcus thermophilus*, *Saccharomyces cerevisiae*, *Enterococcus faecium*, and others [301,302]. These probiotics exert their beneficial effects on the gut microbiota by stimulating the growth of beneficial anaerobic bacteria while suppressing pathogenic and opportunistic microorganisms. This suppression occurs through the production of antibacterial compounds, competition for nutrients and adhesion receptors, and modulation of the ecological balance within the gut [301,303]. By optimising the gut ecosystem, probiotics directly affect mucosal immune mechanisms, i.e., stimulating cytokine synthesis, increasing IgA secretion, promoting phagocytosis, and producing substances that inhibit bacterial virulence factors [294,304]. Together, these mechanisms suppress inflammation and improve the immune response. In addition, probiotics exert trophic effects on the intestinal mucosa by stimulating the proliferation of normal epithelial cells [298]. This activity supports the integrity of the mucosal barrier, which plays a critical role in protecting against microbial translocation and maintaining intestinal homeostasis [305].

**Figure 10:**
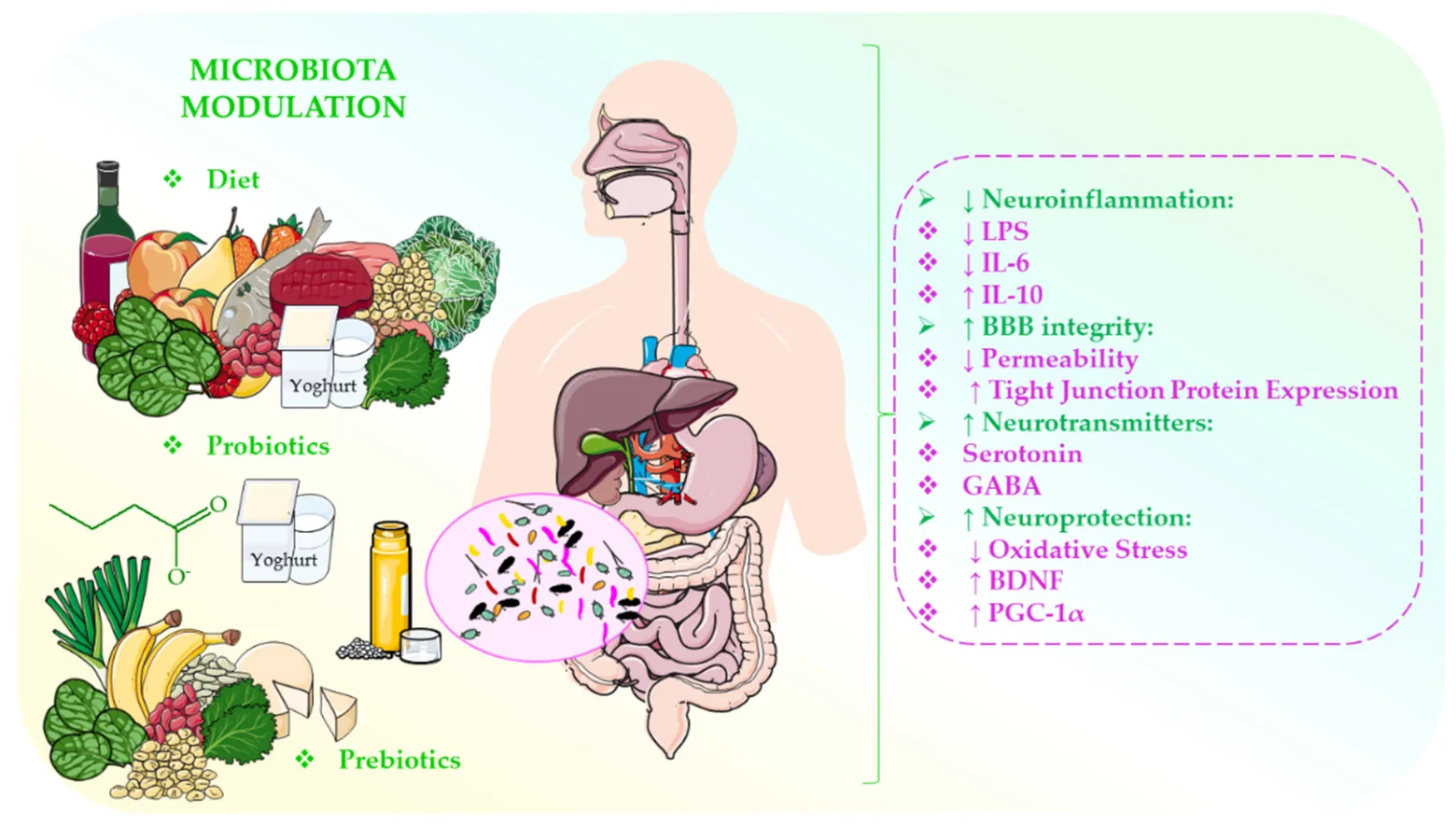
Therapeutic modulation of gut microbiota in neurodegenerative diseases. Modulation of gut microbiota through diet, probiotics, and other therapeutic interventions may represent a promising strategy for alleviating symptoms and slowing the progression of neurodegenerative diseases. These approaches can influence the gut–brain axis, affecting inflammation, metabolic balance, and neuronal function. Abbreviations: BDNF—brain-derived neurotrophic factor; BBB—blood–brain barrier; GABA—gamma-aminobutyric acid; IL-6—interleukin-6; IL-10—interleukin-10; LPS—lipopolysaccharides; PGC-1α—peroxisome; ↑—increase/upregulation; ↓—decrease/downregulation.

Emerging evidence suggests that modulation of the gut microbiota through diet, probiotics, or other therapeutic interventions may offer significant potential as a strategy to alleviate symptoms or slow the progression of neurodegenerative diseases, as shown in Figure 10. However, further studies are needed to fully elucidate the complex mechanisms underlying the gut–brain connection and to explore the therapeutic potential of targeting the gut microbiota in the context of neurodegenerative diseases.

The gut microbiota is increasingly being recognised as a key player in the pathophysiology of neurodegenerative diseases, with several molecular factors and biomarkers emerging as potential links between gut microbial imbalance and neurodegeneration. These factors include microbial metabolites, inflammatory mediators, and alterations in the gut–brain axis, all of which can influence neurodegenerative processes at the molecular level [18]. Therefore, understanding the effect of the modulation of the gut microbiota on the brain could pave the way for novel therapeutic approaches to mitigate or even prevent the progression of neurodegenerative diseases.

### 2.13. Antioxidant-Based Therapies and Gut Microbiota Modulation as a Promising Strategy for the Treatment of Neurodegenerative Diseases

Antioxidant-based therapies have emerged as a promising approach to modulate the gut–brain axis, with a focus on neurodegenerative diseases, such as AD and PD [37,306]. Modulation of the gut microbiota with antioxidants is an important strategy for reducing neuroinflammation and oxidative stress in the brain [37]. A key strategy is the use of antioxidant-enriched probiotics and prebiotics [307]. Probiotics, such as *Lactobacillus* and *Bifidobacterium* strains, promote the growth of beneficial gut bacteria, which in turn produce antioxidant metabolites, such as SCFAs, that help maintain cellular homeostasis [266]. Prebiotics, which serve as nutrient sources for probiotics, are often derived from fibre-rich sources that stimulate the production of antioxidant molecules [308]. By reducing ROS levels both in the gut and systemically, antioxidant-enriched probiotic and prebiotic formulations alleviate neuroinflammation and protect neurons from oxidative damage [309].

Another important approach to using antioxidants to modulate the gut microbiota is through dietary intervention. Foods rich in polyphenols, such as berries, green tea, and dark chocolate, promote the growth of beneficial bacteria while suppressing pathogenic species [310,311,312]. Polyphenols are metabolised by the gut microbiota into bioactive compounds with potent antioxidant properties. These compounds, including phenolic acids and flavonoids, help to restore microbial balance and reduce inflammation in the gut [178,313]. By increasing the gut’s ability to produce natural antioxidants, dietary interventions not only improve gut health but also enhance communication along the gut–brain axis, reducing oxidative stress and neuroinflammation in the brain [37,314].

In addition to dietary strategies, synbiotics—combinations of probiotics and prebiotics with antioxidant-rich components—offer a therapeutic option with a synergistic effect by enhancing the production of important metabolites, e.g., butyrate [315]. Butyrate, i.e., a SCFA with potent anti-inflammatory and antioxidant properties, improves the integrity of the intestinal barrier by reducing intestinal permeability, thereby preventing the leakage of pro-inflammatory molecules into the bloodstream [132]. Synbiotics have been shown to maintain a healthy gut environment, protecting against systemic inflammation and subsequent activation of neuroinflammatory pathways [316]. This dual effect on both the gut and the brain highlights the potential of synbiotics as a comprehensive treatment for neurodegenerative diseases [316,317].

In addition to their direct effects on the gut microbiota, pharmacological approaches targeting antioxidant compounds have also been shown to modulate the gut–brain axis. This includes the use of such molecules as resveratrol, curcumin, vitamin C, vitamin E, and β-carotene, which have been shown to modulate the composition and function of the gut microbiota by reducing ROS levels in the gut and increasing the expression of antioxidant enzymes, such as glutathione peroxidase, in intestinal cells [318,319,320]. By stabilising gut microbial communities and reducing oxidative stress, these pharmacological agents indirectly support brain health, thereby mitigating the progression of neurodegenerative diseases [37,177,314].

In summary, the presented research field is moving towards the development of personalised therapeutic regimens, with a focus on optimising antioxidant-based interventions tailored to an individual’s unique gut microbiota composition and metabolic needs. Recent advances in microbiome research and genetic analysis facilitate the development of precision dietary interventions, including personalised dietary plans, probiotic formulations, and antioxidant supplements [321,322]. These interventions are designed to target specific microbial imbalances or oxidative stress pathways, maximising therapeutic efficacy and offering potential relief to individuals affected by neurodegenerative diseases.

## 3. Conclusions

The importance of microbial metabolites, such as SCFAs, in the regulation of systemic and neuroinflammation, neurotransmitter synthesis, and hormonal stress response via the HPA axis further highlights the link between the gut microbiota and neurodegenerative diseases, including AD, PD, and ALS. SCFAs produced by gut microbes play a critical role in maintaining the integrity of the blood–brain barrier and modulating immune responses, which may help prevent or alleviate neurodegenerative symptoms. In addition, the gut–brain axis regulates the synthesis of neurotransmitters, e.g., serotonin, through tryptophan metabolism, affecting mood and cognitive function, which are often impaired in neurodegenerative diseases. This highlights the significant and far-reaching impact of interventions aimed at modulating the composition of the gut microbiota on brain health.

Modulation of the gut microbiota through dietary interventions, probiotics, and synbiotics (probiotics combined with prebiotics and antioxidant-rich compounds) represents a promising strategy for preventing or slowing disease progression. Such interventions can promote a healthy gut environment, alleviate oxidative stress, and potentially restore the balance of neuroactive compounds, providing a multi-targeted approach to combat neurodegeneration. Modulation of the vagus nerve, a central mediator in the gut–brain axis, and the effects of gut-derived metabolites on inflammation, neurotransmitter synthesis, and immune regulation offer novel therapeutic opportunities. In addition, antioxidant-based therapies may have synergistic effects when combined with microbiome-based strategies, enhancing their overall efficacy. Targeting the gut–brain axis through antioxidant therapies and microbiome modulation has emerged as a promising approach to mitigate neurodegeneration, reduce chronic inflammation, and improve patient outcomes in AD, PD, and other neurodegenerative diseases. This integrated approach has the potential to advance the prevention, early intervention, and management of these debilitating conditions.

Recent studies have demonstrated the ability of antioxidants to influence the composition of the gut microbiome, reduce oxidative stress, and modulate key molecular pathways, such as NF-κB, Nrf2, MAPK and PI3K/AKT, involved in neurodegeneration. By targeting oxidative stress and restoring the balance within the gut microbiota, antioxidants may reduce the inflammatory milieu that exacerbates such diseases as AD and PD. There is also evidence that antioxidants can increase microbial diversity, which could benefit brain health by reducing neuroinflammation and promoting neuroprotective effects.
