# Correction: Kurhaluk et al. Role of Antioxidants in Modulating the Microbiota–Gut–Brain Axis and Their Impact on Neurodegenerative Diseases. *Int. J. Mol. Sci.* 2025, *26*, 3658

**DOI:** 10.3390/ijms27125214

**Published:** 2026-06-09

**Authors:** Natalia Kurhaluk, Piotr Kamiński, Rafał Bilski, Renata Kołodziejska, Alina Woźniak, Halina Tkaczenko

**Affiliations:** 1 Institute of Biology, Pomeranian University in Słupsk, Arciszewski St. 22 B, 76-200 Słupsk, Poland; halina.tkaczenko@upsl.edu.pl; 2 Department of Medical Biology and Biochemistry, Division of Ecology and Environmental Protection, Collegium Medicum in Bydgoszcz, Nicolaus Copernicus University in Toruń, M. Skłodowska-Curie St. 9, 85-094 Bydgoszcz, Poland; piotr.kaminski@cm.umk.pl; 3 Department of Biotechnology, Institute of Biological Sciences, Faculty of Biological Sciences, University of Zielona Góra, Prof. Z. Szafran St. 1, 65-516 Zielona Góra, Poland; 4 Department of Medical Biology and Biochemistry, Collegium Medicum in Bydgoszcz, Nicolaus Copernicus University in Toruń, M. Karłowicz St. 24, 85-092 Bydgoszcz, Poland; rafal.bilski@cm.umk.pl (R.B.); renatak@cm.umk.pl (R.K.); al1103@cm.umk.pl (A.W.)

## Error in Figure

In the original publication [1], there was a mistake in Figures 1–10 as published. Following editorial assessment, Figures 1–10 in the original publication [1] have been replaced with newly prepared versions created by the authors. The revised Figure 1, Figure 2, Figure 3, Figure 4, Figure 5, Figure 6, Figure 7, Figure 8, Figure 9 and Figure 10 include improvements in resolution, labeling, consistency, and overall clarity. No changes affecting the scientific content, interpretation, or conclusions of the manuscript have been introduced.

## Reference Correction

After the publication of our review article [1], one of the referenced manuscripts was found to have been retracted. To enhance the quality of the manuscript, we decided to replace the previous reference [186] in the original manuscript with a new reference in the same position in the reference list. This replacement does not affect the meaning of the original text in the published paper. There was no need to adjust the number for each cited reference in the References section.

The original reference is as follows: Ashique, S.; Mohanto, S.; Ahmed, M.G.; Mishra, N.; Garg, A.; Chellappan, D.K.; Omara, T.; Iqbal, S.; Kahwa, I. Gut-brain axis: A cutting-edge approach to target neurological disorders and potential synbiotic application. *Heliyon* **2024**, *10*, e34092.

The new replacement for reference [186] is as follows: Kurhaluk, N.; Kołodziejska, R.; Kamiński, P.; Tkaczenko, H. Integrative Neuroimmune Role of the Parasympathetic Nervous System, Vagus Nerve and Gut Microbiota in Stress Modulation: A Narrative Review. *Int. J. Mol. Sci.* **2025**, *26*, 11706. https://doi.org/10.3390/ijms262311706.

The authors state that the scientific conclusions are unaffected. This correction was approved by the Academic Editor. The original publication has also been updated.

## Figures and Tables

**Figure 1:**
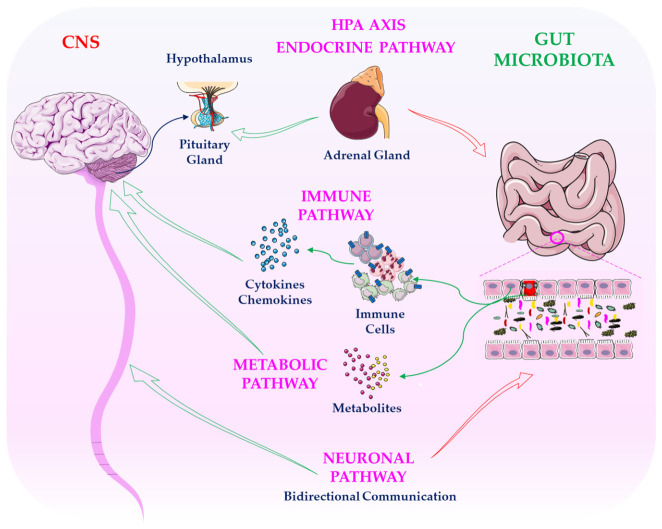
Bidirectional communication in the microbiome–brain axis. Bidirectional signalling within the microbiome–brain axis involves complex neural, endocrine, immune, and metabolic pathways, enabling dynamic crosstalk between the gut microbiota and the central nervous system. These interactions influence neurodevelopment, behaviour, and host physiology, highlighting the therapeutic potential of microbiota-targeted interventions in neurodegenerative diseases. Image provided by Servier Medical Art (https://smart.servier.com), licensed under CC BY 4.0 (https://creativecommons.org/licenses/by/4.0/, accessed on 26 March 2026). Abbreviations: CNS—central nervous system; HPA Axis—hypothalamic–pituitary–adrenal axis.

**Figure 2:**
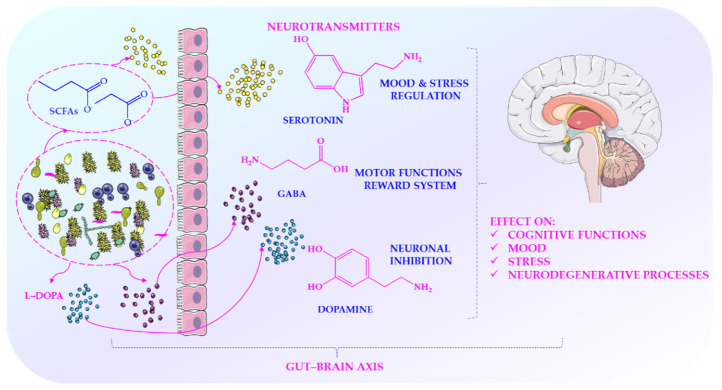
Gut Microbiota–mediated modulation of neurotransmitter synthesis and its impact on brain function and neurodegeneration. Role of the gut microbiota in regulating the synthesis and metabolism of key neurotransmitters (serotonin, dopamine, and GABA) and its impact on mood, cognitive function, and neurodegenerative processes. Alterations in gut microbial composition may disrupt the gut–brain axis, contributing to mood disorders and the progression of neurological diseases. Image provided by Servier Medical Art (https://smart.servier.com), licensed under CC BY 4.0 (https://creativecommons.org/licenses/by/4.0/, accessed on 26 March 2026). Abbreviations: GABA—gamma-aminobutyric acid; L-DOPA—L-3,4-dihydroxyphenylalanine; SCFAs—short-chain fatty acids.

**Figure 3:**
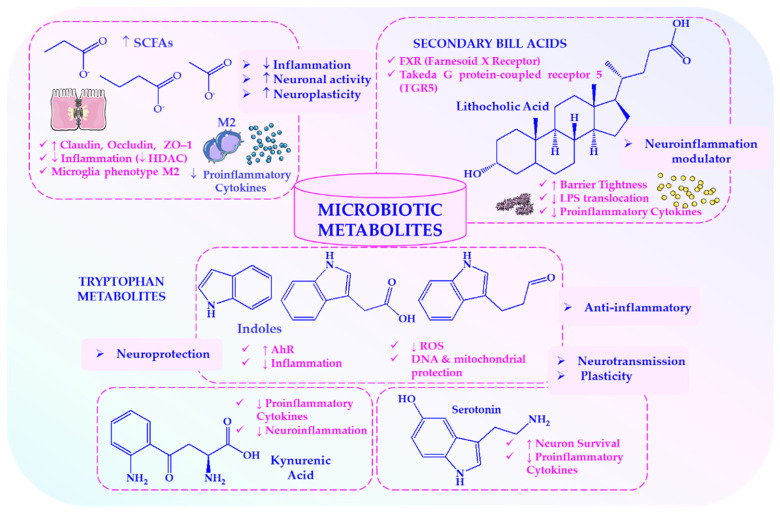
Metabolic pathways of the gut microbiota and their relevance to neurophysiology. Interactions between key microbial metabolic pathways include the role of short-chain fatty acids (SCFAs) in neurotransmission, tryptophan metabolism via the kynurenine pathway, and bile acid and lipid metabolism. Microbial metabolites such as SCFAs, tryptophan derivatives, and bile acids influence brain function through complex metabolic and signaling mechanisms, modulating neuroinflammation, immune responses, and the gut–brain axis. Image provided by Servier Medical Art (https://smart.servier.com), licensed under CC BY 4.0 (https://creativecommons.org/licenses/by/4.0/, accessed on 26 March 2026). Abbreviations: AhR—aryl hydrocarbon receptor; DNA—deoxyribonucleic acid; FXR—farnesoid X receptor; HDAC—histone deacetylases; LPS—lipopolysaccharide; M2—M2 macrophages; ROS—reactive oxygen species; SCFAs—short-chain fatty acids; TGR5—Takeda G protein-coupled receptor 5; ZO-1—zonula occludens-1; ↑—increase/upregulation; ↓—decrease/downregulation.

**Figure 4:**
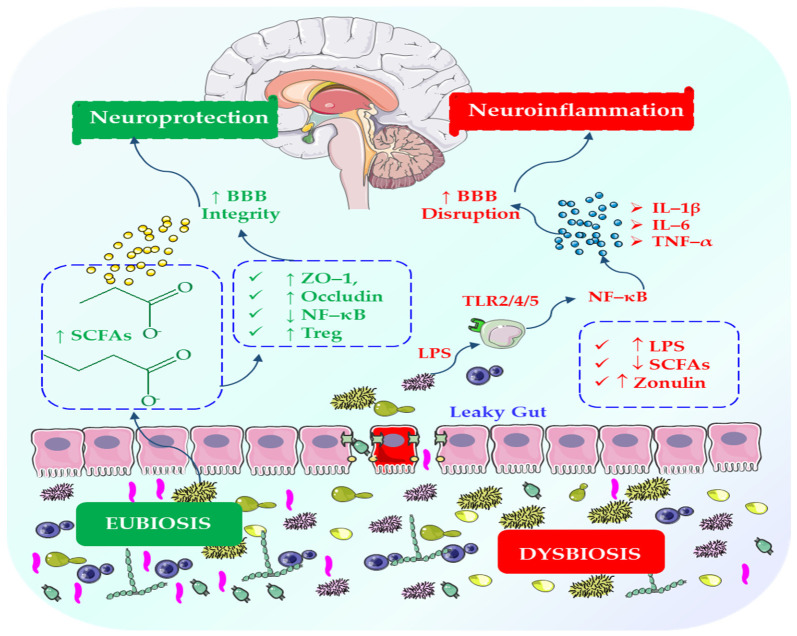
Immune system pathways. The gut microbiota play a crucial role in modulating both systemic and neuroinflammatory responses. The influence of the gut microbiota on immune signalling is achieved through cytokine production, regulation of blood–brain barrier (BBB) permeability, and interactions with key immune biomarkers and molecular pathways. These pathways underscore the intricate communication between gut-resident microbes, immune cells, and the central nervous system, thereby underscoring the significance of microbiota in maintaining immune homeostasis and potentially contributing to the pathophysiology of neurological and inflammatory disorders. Image provided by Servier Medical Art (https://smart.servier.com), licensed under CC BY 4.0 (https://creativecommons.org/licenses/by/4.0/, accessed on 26 March 2026). Abbreviations: BBB—blood—brain barrier; IL-1β—interleukin-1 beta; IL-6—interleukin-6; LPS—lipopolysaccharide; NF-κB—nuclear factor kappa-light-chain-enhancer of activated B cells; SCFAs—short-chain fatty acids; TLR 2/4/5—Toll-like receptors 2, 4, and 5; TNF-α—tumor necrosis factor alpha; Treg—regulatory T cells; ZO-1—zonula occludens-1, ↑—increase/upregulation; ↓—decrease/downregulation.

**Figure 5:**
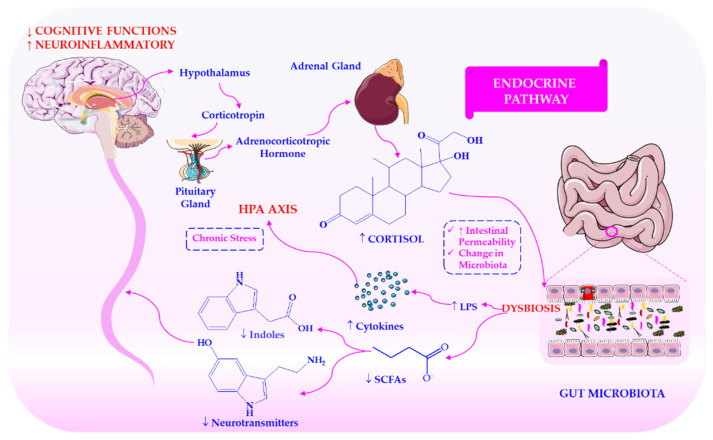
Gut microbiota in the regulation of the HPA axis and neuroendocrine signaling. The gut microbiota modulates the hypothalamic–pituitary–adrenal (HPA) axis, influencing stress, appetite, and behavior. Intestinal microorganisms regulate cortisol and ACTH secretion and synthesize neurotransmitters, as well as cytokines and indoles, thereby affecting mood and cognitive functions. Disruption of gut homeostasis may lead to HPA axis dysregulation and hormonal imbalance. Image provided by Servier Medical Art (https://smart.servier.com), licensed under CC BY 4.0 (https://creativecommons.org/licenses/by/4.0/, accessed on 26 March 2026). Abbreviations: HPA Axis—hypothalamic–pituitary–adrenal axis; LPS—lipopolysaccharides; SCFAs—short-chain fatty acids, ↑—increase/upregulation; ↓—decrease/downregulation.

**Figure 6:**
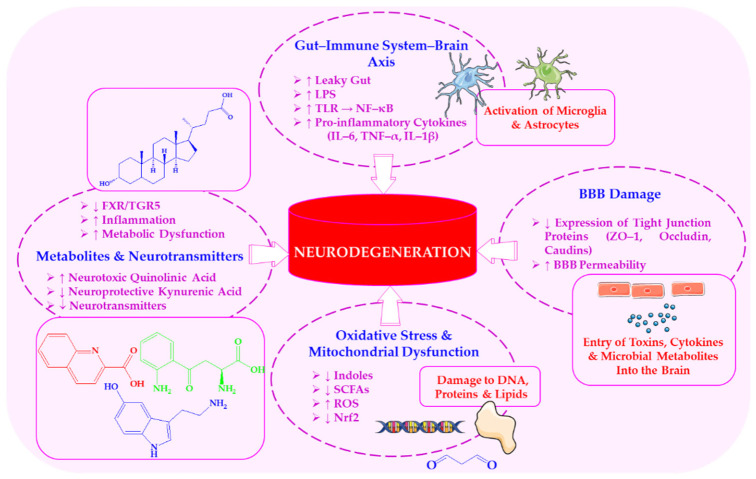
Molecular mechanisms linking gut microbiota to neurodegeneration. Gut microbiota influence neurodegeneration by promoting chronic inflammation, impairing the blood–brain barrier, altering neurotransmitters production, disrupting gut–brain axis communication, and contributing to oxidative stress and mitochondrial dysfunction. Image provided by Servier Medical Art (https://smart.servier.com), licensed under CC BY 4.0 (https://creativecommons.org/licenses/by/4.0/, accessed on 26 March 2026). Abbreviations: BBB—blood–brain barrier; DNA—deoxyribonucleic acid; FXR—farnesoid X receptor; IL-1β—interleukin-1 beta; IL-6—interleukin-6; LPS—lipopolysaccharides; NF-κB— nuclear factor kappa-light-chain-enhancer of activated B cells; Nrf2—nuclear factor erythroid 2-related factor 2; ROS—reactive oxygen species; SCFAs—short-chain fatty acids; TGR5—Takeda G-protein-coupled receptor 5; TLR—toll-like receptor; TNF-α—tumor necrosis factor alpha; ZO-1—zonula occludens-1; ↑—increase/upregulation; ↓—decrease/downregulation.

**Figure 7:**
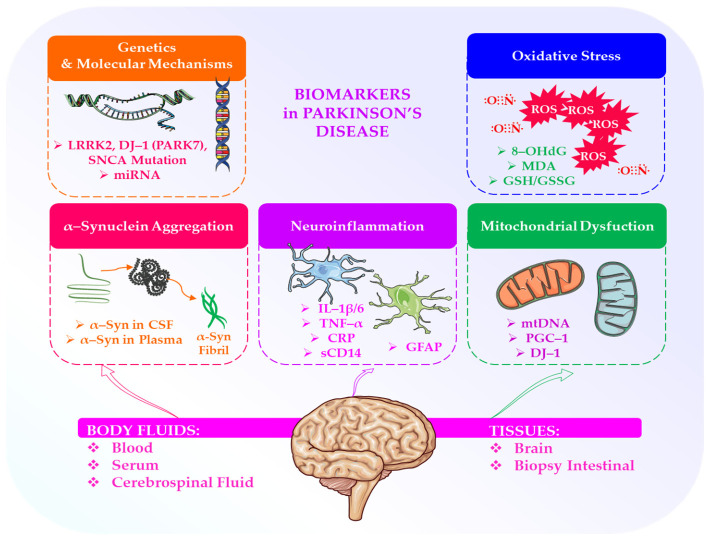
Biomarkers associated with molecular pathways in Parkinson’s disease. In Parkinson’s disease (PD), biomarkers are recognized as indicators of key molecular processes, including genetic and signaling pathways, α-synuclein aggregation, neuroinflammation, mitochondrial dysfunction, and oxidative stress. These interconnected mechanisms drive disease progression and are reflected by specific biomarkers that can be detected in body fluids and neural tissue. Image provided by Servier Medical Art (https://smart.servier.com), licensed under CC BY 4.0 (https://creativecommons.org/licenses/by/4.0/, accessed on 26 March 2026). Abbreviations: 8-OHdG—8-Hydroxy-2′-deoxyguanosine; α-Syn—alpha-synuclein; CRP—C-reactive protein; CSF—cerebrospinal fluid; DJ-1 (PARK7)—protein deglycase DJ-1; GSH—reduced glutathione; GSSG—oxidized glutathione; GFAP—glial fibrillary acidic protein; IL-1β—interleukin-1 beta; IL-6—interleukin-6; LRRK2—leucine-rich repeat kinase 2; MDA—malondialdehyde; mtDNA—mitochondrial DNA; NO—nitric oxide; PGC-1α—peroxisome; ROS—reactive oxygen species; sCD14—soluble CD14; SNCA—synuclein alpha; TNF-α—tumor necrosis factor alpha.

**Figure 8:**
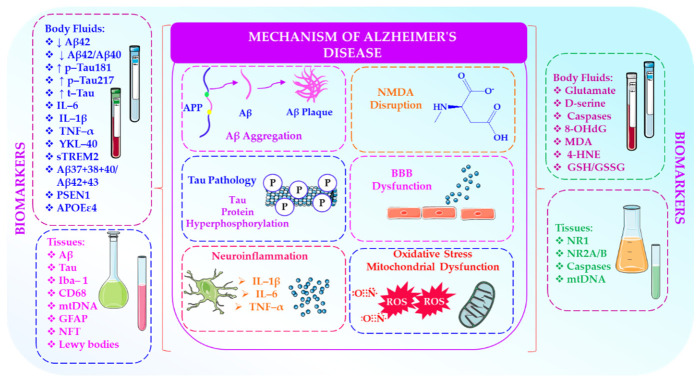
Mechanisms, biomarkers and molecular pathways of Alzheimer’s disease. Alzheimer’s disease is a multifactorial disorder characterized by amyloid-β (Aβ) accumulation, tau pathology, neuroinflammation, mitochondrial dysfunction, and vascular alterations. Increasing evidence highlights the role of the gut microbiota in modulating these processes, including Aβ aggregation, blood–brain barrier integrity, and oxidative stress. Associated biomarkers, such as inflammatory cytokines, tight junction proteins, and oxidative stress markers, can be detected in body fluids and tissues, providing valuable insights into disease mechanisms, diagnosis, and progression. Abbreviations: 4-HNE—4-hydroxynonenal; 8-OHdG—8-hydroxy-2′-deoxyguanosine; Aβ—amyloid-β; Aβ37+38+40/Aβ42+43—composite amyloid-β peptide ratios; Aβ42—amyloid-β 42; Aβ42/Aβ40—amyloid-β ratio; APOEε4—apolipoprotein E epsilon 4; APP—amyloid precursor protein; BBB—blood–brain barrier; CD68—a marker of activated microglia and macrophages; GFAP—glial fibrillary acidic protein; GSH—reduced glutathione; GSH/GSSG—glutathione ratio; GSSG—oxidized glutathione; Iba-1—ionized calcium-binding adapter molecule 1; IL-1β—interleukin-1 beta; IL-6—interleukin-6; MDA—malondialdehyde; mtDNA—mitochondrial DNA; NFT—neurofibrillary tangles; NMDA—N-methyl-D-aspartate receptor; NO—nitric oxide; NR1—NMDA receptor subunit NR1; NR2A/B—NMDA receptor subunits NR2A and NR2B; p-Tau181—phosphorylated Tau at threonine 181; p-Tau217—phosphorylated Tau at threonine 217; PSEN1—presenilin-1; ROS—reactive oxygen species; sTREM2—soluble triggering receptor expressed on myeloid cells 2; Tau—microtubule-associated protein Tau; TNF-α—tumor necrosis factor alpha; YKL-40—chitinase-3-like protein 1; ↑—increase/upregulation; ↓—decrease/downregulation.

**Figure 9:**
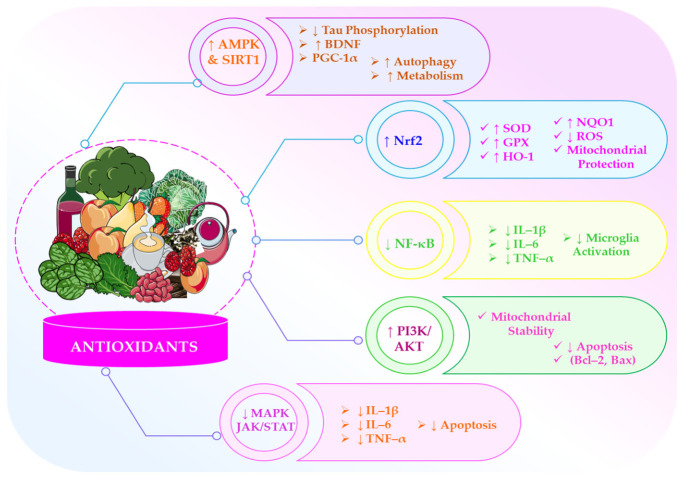
Antioxidant-mediated modulation of molecular pathways in neurodegenerative diseases. Molecular mechanisms and signaling pathways illustrate how antioxidants influence key processes involved in neurodegenerative diseases, providing protection against oxidative stress, neuroinflammation, and cellular damage. Antioxidants can modulate mitochondrial function, reduce reactive oxygen species production, and regulate inflammatory signaling pathways, thereby preserving neuronal integrity and function. Abbreviations: AKT—protein kinase B; AMPK—AMP-activated protein kinase; Bax—Bcl-2-associated X protein; BDNF—brain-derived neurotrophic factor; Bcl-2—B-cell lymphoma 2; GPX—glutathione peroxidase; HO-1—heme oxygenase-1; IL-1β—interleukin-1 beta; IL-6—interleukin-6; JAK—Janus kinase; MAPK—mitogen-activated protein kinase; NF-κB —nuclear factor kappa-light-chain-enhancer of activated B cells; NQO1—NAD(P)H quinone dehydrogenase 1; Nrf2—nuclear factor erythroid 2-related factor 2; PGC-1α—peroxisome; PI3K—phosphoinositide 3-kinase; ROS—reactive oxygen species; SIRT1—sirtuin 1; SOD—superoxide dismutase; STAT—signal transducer and activator of transcription; Tau—microtubule-associated protein Tau; TNF-α—tumor necrosis factor alpha; ↑—increase/upregulation; ↓—decrease/downregulation.

**Figure 10:**
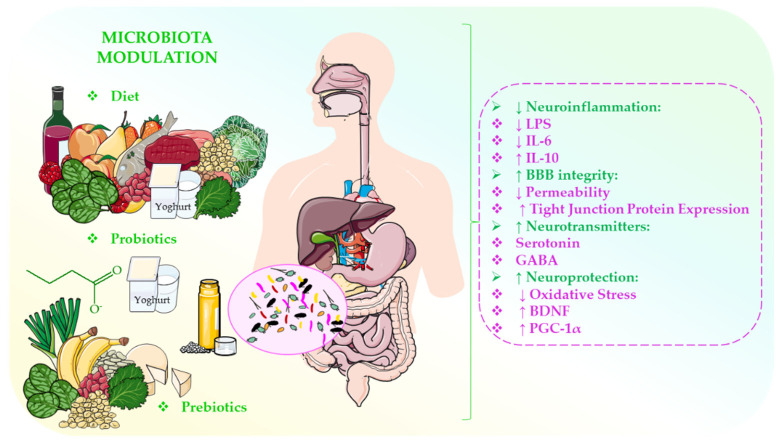
Therapeutic modulation of gut microbiota in neurodegenerative diseases. Modulation of gut microbiota through diet, probiotics, and other therapeutic interventions may represent a promising strategy for alleviating symptoms and slowing the progression of neurodegenerative diseases. These approaches can influence the gut–brain axis, affecting inflammation, metabolic balance, and neuronal function. Abbreviations: BDNF—brain-derived neurotrophic factor; BBB—blood–brain barrier; GABA—gamma-aminobutyric acid; IL-6—interleukin-6; IL-10—interleukin-10; LPS—lipopolysaccharides; PGC-1α—peroxisome; ↑—increase/upregulation; ↓—decrease/downregulation.

## References

[1] Kurhaluk N., Kamiński P., Bilski R., Kołodziejska R., Woźniak A., Tkaczenko H. (2025). Role of Antioxidants in Modulating the Microbiota–Gut–Brain Axis and Their Impact on Neurodegenerative Diseases. Int. J. Mol. Sci..

